# Periodic, Quasi-periodic and Chaotic Dynamics in Simple Gene Elements with Time Delays

**DOI:** 10.1038/srep21037

**Published:** 2016-02-15

**Authors:** Yoko Suzuki, Mingyang Lu, Eshel Ben-Jacob, José N. Onuchic

**Affiliations:** 1 Department of Physics, School of Science and Engineering, Meisei University, 2-1-1 Hodokubo, Hino-shi, Tokyo 191-8506, Japan; 2 Center for Theoretical Biological Physics, Rice University, Houston, TX 77005-1827, USA; 3 Department of Physics and Astronomy, Rice University, Houston, TX 77005-1827, USA; 4 Department of Chemistry, Rice University, Houston, TX 77005-1827, USA; 5 Department of Biochemistry and Cell Biology, Rice University, Houston, TX 77005-1827, USA; 6 School of Physics and Astronomy and The Sagol School of Neuroscience, Tel-Aviv University, Tel-Aviv 69978, Israel

## Abstract

Regulatory gene circuit motifs play crucial roles in performing and maintaining vital cellular functions. Frequently, theoretical studies of gene circuits focus on steady-state behaviors and do not include time delays. In this study, the inclusion of time delays is shown to entirely change the time-dependent dynamics for even the simplest possible circuits with one and two gene elements with self and cross regulations. These elements can give rise to rich behaviors including periodic, quasi-periodic, weak chaotic, strong chaotic and intermittent dynamics. We introduce a special power-spectrum-based method to characterize and discriminate these dynamical modes quantitatively. Our simulation results suggest that, while a single negative feedback loop of either one- or two-gene element can only have periodic dynamics, the elements with two positive/negative feedback loops are the minimalist elements to have chaotic dynamics. These elements typically have one negative feedback loop that generates oscillations, and another unit that allows frequent switches among multiple steady states or between oscillatory and non-oscillatory dynamics. Possible dynamical features of several simple one- and two-gene elements are presented in details. Discussion is presented for possible roles of the chaotic behavior in the robustness of cellular functions and diseases, for example, in the context of cancer.

One of the challenges in molecular cell biology is to understand how cells fulfill their functions through specific gene regulations^1^. Various state-of-art experimental techniques, such as high-throughput DNA/RNA sequencing^2^ and whole-cell genomic/proteomic profiling^3^, have been developed to enable the mapping or the inference of gene regulatory network^4^,^5^. Yet, it remains unclear how cells utilize the gene network to perform their specific tasks and to do so efficiently despite the innate high intracellular noise^6^,^7^,^8^,^9^.

Many theoretical and synthetic biology studies have suggested that a specific set of topological links among a few genes, namely circuit motifs, may individually perform certain functions^10^,^11^,^12^,^13^. For instance, a toggle switch (two genes mutual-inhibiting each other) and its variations allow the coexistence of multiple stable steady states^14^,^15^, which is essential to decision-making between cellular fates during cell differentiation^16^,^17^ and certain cell phenotypic transitions, such as epithelial-to-mensenchymal transitions^18^,^19^. Motifs such as flip-flop circuit^20^ and repressilator circuit^21^ have been shown to allow periodic oscillations in gene expression levels. Moreover, a feed forward loop may generate pulses, detect fold-changes or make adaptation in response to different external and internal signals^22^,^23^.

It is commonly assumed that these circuit motifs are the building block modules of a larger modular network that can perform several elaborated tasks as needed. For the modular network to function efficiently, the individual circuit modules should have the following properties. First, each circuit should have sufficient functional flexibility to perform its specific function while it receives various inputs from the other modules in the network. Second, the module dynamical behavior should be stable in the presence of internal and external noise. Third, the module function should be robust to changes in the circuit parameters that vary from cell to cell. The current study is motivated by the need to investigate the sensitivity to the circuit parameters. We seek to check under which circumstances the circuit dynamics can be dramatically different, or even become chaotic for a certain range of parameters.

In general, the dynamics of gene expression for a gene circuit can be modeled by coupled nonlinear ordinary differential equations. Thus, chaotic behavior could theoretically exist in motifs comprised of three or more components (e.g. three genes) or whose dynamics is described by three or more equations^24^. Chaotic behavior in gene circuits has been studied before^20^,^25^,^26^,^27^,^28^,^29^,^30^, but it has gained limited attention in systems biology for the following reasons. First, it is commonly assumed that the selected gene networks, during the course of evolution, are those that are robust to noise and changes in the circuit dynamics. Second, it is very hard to quantitatively measure chaotic dynamics of gene expression due to the limited availability of temporal gene expression data and due to the presence of gene expression noises in circuit dynamics. Third, it has been shown in computational studies that chaos motifs are rare and the parameter range to observe the chaotic dynamics for such motifs is extremely narrow^25^.

We propose that when time delays are included, chaotic dynamics could be observed even in simple circuit elements with one or two elements and for a much wider range of circuit parameters than previously expected. Chaos is observed even for two coupled genes with time delayed mutual regulations or even a single gene with two time delayed self-regulations. Moreover, we reason that chaotic dynamics can be very relevant in abnormal physiological conditions and in some diseases, such as cancer, where gene regulations and circuit parameters significantly differ from the evolutionary selected ones. Hence, different from previous studies of chaotic dynamics in gene circuits, we now include the effects of time delays in the self and cross regulations. Time delays in gene regulations may arise from the recruitment of RNA polymerase, transportation of mRNAs and translational process through ribosome, and several other sources in the cell. While time delays do not change the steady states solutions of the gene networks, they can change the stability of these states and the circuits’ dynamical behaviors^31^,^32^,^33^. Therefore in this study we show that time delays can add singularity to the nonlinear dynamics of gene expression. We note for completeness that in a different context some previous studies in an homogeneous population of circulating white blood cells have shown that inclusion of time delays can give rise to chaotic dynamics^34^.

As it is mentioned earlier, this paper investigates the dynamics of simple one and two gene circuit motifs when time delays are included. In the next section, we present a concise description of the circuit motifs and their possible dynamical behaviors. We show that these circuits give rise to a rich variety of dynamical modes-periodic (P), quasi-periodic (QP), weak chaotic (WC) and strong chaotic (SC) dynamics. Over the years, researchers have been characterizing the properties of chaotic behaviors and distinguishing between the different types of chaos by various methods and criteria^24^, including power spectrum, Lorenz map^35^, features of a trajectory in the phase space^36^ such as strange attractor^37^, Poincare map^38^, Lyapunov stability^39^,^40^ and Grassberger-Procaccia algorithm^41^. Yet, different authors use different terminology to describe the various chaotic behaviors^42^. For example, some use the terms chaos and hyperchaos to describe weak chaos and strong chaos^43^,^44^. Here we introduce a special power-spectrum-based method to distinguish among the various dynamical modes in the gene circuits that we have studied.

As elaborated in details in the results section, we found that even the simple one and two gene motifs, that are frequently recurring in most biological networks, are capable of generating P, QP, WC and SC dynamics for a wide range of time delays and circuit parameters. Moreover, transitions from non-chaotic dynamics (presumably corresponding to normal cells) to more elaborated dynamics (presumably in abnormal cells) only require relatively small changes in the time delays. Interestingly, for some circuits that contain both positive and negative feedback loops, the circuits can give rise to intermittent dynamics between two modes (e.g. P-SC, and WC-SC). At the end of the article, we discuss possible roles of the chaotic behavior in the robustness of cellular functions and their relevance to some diseases.

## Roadmap

**Figure 1:**
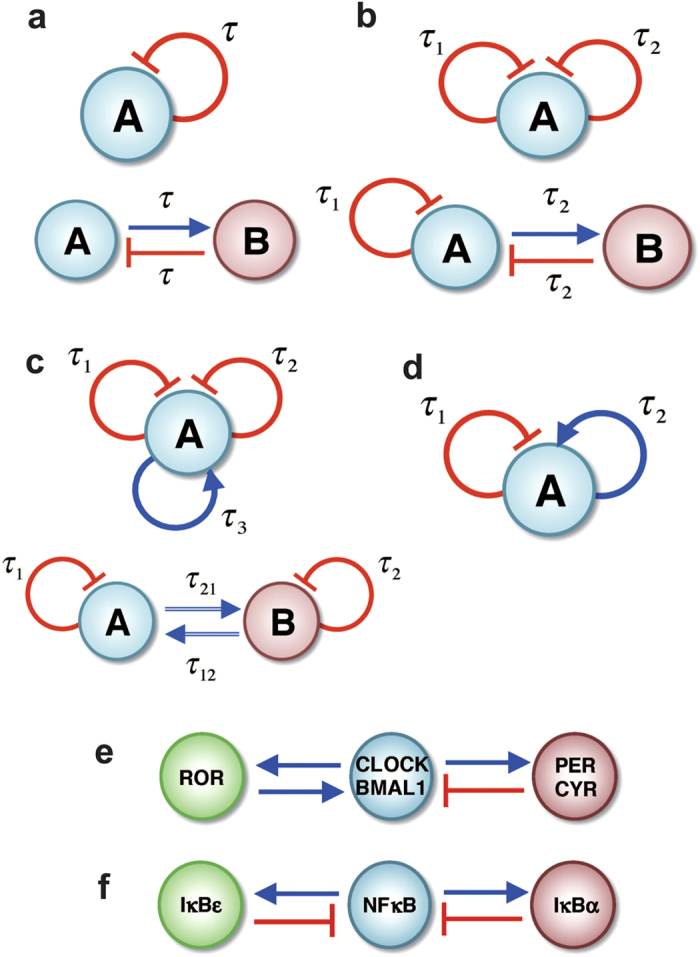
A list of circuit motifs used in this study. The light blue and light red circles represent genes A and B, respectively. The blue lines with arrows represent transcriptional activation and the red lines with bars represent transcriptional inhibition. A symbol τ along each line represents the time delay in the interaction. In the Results, we show that different groups of circuit motifs exhibit unique dynamics. (**a**) shows motifs with a negative feedback loop. These one-gene and two-gene elements exhibit periodic oscillations. (**b**) shows motifs with two negative feedback loops. They exhibit periodic, quasi-periodic and weak chaotic dynamics. (**c**) shows motifs with two negative feedback loops and a positive feedback loop. They exhibit periodic, quasi-periodic, weak chaotic, and strong chaotic dynamics. (**d**) shows a one-gene element with both self-activation and self-inhibition. For certain parameter range, the motif may exhibit intermittent dynamics between periodic and weak chaotic mode. Intermittency between weak chaotic and strong chaotic mode was also found for the circuit motif in the upper panel of (**c**). Panel (**e**) and (**f**) show the core circadian clock gene circuit and the NFκB gene circuit respectively.

In this section, we discuss the circuit motifs whose dynamics are studied in this paper (Fig. 1). They are among the simplest possible circuit motifs, consisting of either one gene or two genes. Yet, when considering time delays in the regulation, we found that even such simple circuits exhibit rich dynamical behaviors. To classify these dynamics, especially those non-periodic oscillatory ones, we introduce a special power-spectrum-based method that is used throughout the work. The detailed analyses for each motif is shown in the following sections and in Supplementary Information.

We begin with the simplest gene elements that can give rise to periodic oscillations, since such feature is a prerequisite to have non-periodic oscillations. One of the simplest oscillatory circuit motifs is repressilator. This element, which was first studied as a synthetic circuit^21^ and was later shown to be existent in naturally occurred gene network^45^,^46^, is comprised of three genes with sequential inhibitions (A inhibits B, B inhibits C and C inhibits A). A repressilator is able to generate stable oscillations without introducing time delays in the repressive regulations. In Supplementary section SI1, we show that the dynamics of a repressilator is comparable to that of a self-inhibitory single gene element (Fig. 1a) with time delay. Similar time dynamics could also be observed in a flip-flop circuit with time delays^20^,^31^. Here, the flip-flop motif is a two-gene element (genes denoted by A and B) where A activates B and B inhibits A.

Next, we hypothesized that chaotic gene elements could be built on the basis of these minimalist oscillatory elements and demonstrate that this is indeed the case. We learned that to generate chaos in the gene expression, the elements must have at least two coupled feedback loops with different time delays – either the elements with at least two negative feedback loops (Fig. 1b), or the elements with at least one negative and one positive feedback loops (Fig. 1c,d). In addition, strong chaotic behavior (as explained and defined later) was found in elements with more than two coupled regulatory motifs. A typical example is the elements in Fig. 1c with two coupled negative feedback loops and one positive feedback loop. Note that in all these cases, we found similar non-periodic dynamics for both single-gene and the corresponding two-gene elements, suggesting that it is the coupled circuit motif rather than the number of genes that gives origin to the different chaotic behaviors.

**Figure 2:**
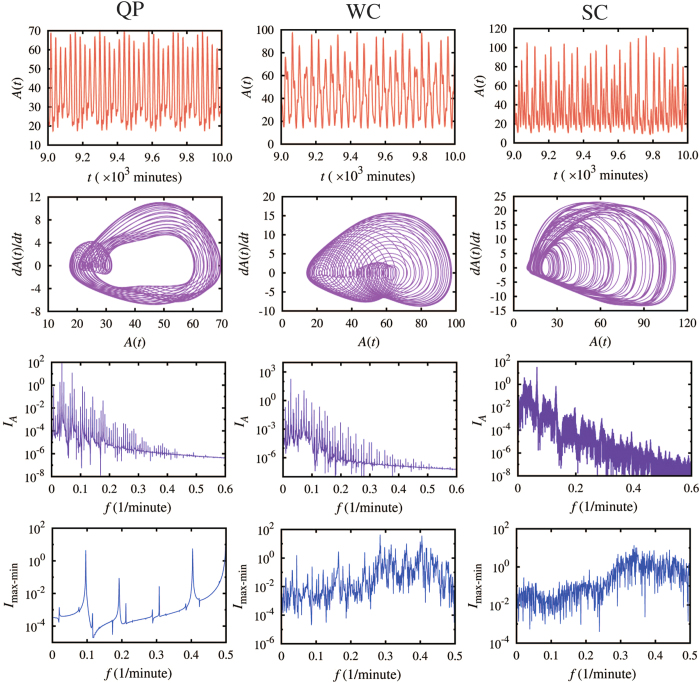
A quantitative description of the differences between various non-periodic dynamical modes. The left, middle and right columns exemplify typical dynamics of quasi-periodic (QP), weak chaotic (WC), and strong chaotic (SC) behaviors, respectively. All the different dynamic regimes can be differentiated qualitatively by the analysis illustrated in the figure. The QP and SC regimes are illustrated by the dynamics of a one-gene element with two delayed self-inhibitions and one delayed self-activation (diagram in the upper panel of Fig.1c); the WC regime is illustrated by the dynamics of a one-gene element with two delayed self-inhibitions (diagram in the upper panel of Fig.1b). The first row shows time trajectories; the second row shows phase-space maps for the expression level A(t) (x-axis) vs. its time derivative dA(t)/dt (y-axis). The map for QP has a torus structure, while those for WC and SC are more irregular. The third row shows the full spectra; the power spectra of the whole time trajectory. The full spectra for the QP and WC cases have sharp peaks at some discrete frequencies, while those for the SC cases have spikes through almost the whole frequency range. The forth row shows the maximum-minimum spectra; the power spectra of the corresponding discrete series that are composed of the maximum and the minimum expressions of every oscillation. The maximum-minimum spectra for the QP cases have sparse spikes only at a few frequencies. Those for the WC and SC cases have spikes through the whole frequency range. But the WC and SC cases are different in that the WC spectra have balanced upward and downward spikes, and the SC spectra have dominant downward spikes. A combination of both the full spectrum and the maximum-minimum spectrum is a powerful tool to discriminate different non-periodic dynamics.

We define the different chaotic behaviors by examining the power spectra of the time trajectories of the gene expression. We utilize two kinds of spectra – the spectrum of the whole time trajectories of the dynamics (termed *full spectrum*) and the spectrum of a corresponding discrete series that is composed of the maximum and the minimum expressions of every oscillation (termed *maximum-minimum spectrum*). By using the combination of the two spectra, we were able to recognize different non-periodic oscillatory dynamics. For example, Fig. 2 shows three different dynamics, representing the typical cases of quasi-periodic (QP, the left column), weak chaotic (WC, the middle column) and strong chaotic (SC, the right column) behaviors. The time trajectories for all these cases look very similar (Fig. 2, the first row). In terms of the full spectrum (Fig. 2, the third row), those for the QP and WC cases are similar since both have spikes at some discrete frequencies. Yet, the full spectrum of the SC case is distinct; it is marked by spikes through almost the whole frequency range. For the maximum-minimum spectrum (Fig. 2, the fourth row), those for the WC and the SC cases are similar since both have many spikes through the whole frequency range. We also noted that the SC spectrum is dominant by the downward spikes, while the WC spectrum has balanced upward and downward spikes. The maximum-minimum spectrum for the QP case is distinct; it is marked by the existence of only a few spikes and they are mostly upward. Another powerful tool is to visualize the time trajectory by projecting it onto a two-dimensional phase space. Here we show the space by the gene expression level vs. the expression time derivative (Fig. 2, the second row). The QP map has a torus structure; the WC map covers the phases more but regularly, while the SC map is more irregular. Note that the combination of these tools also enables us to identify the intermittent P-SC (the circuit in Fig. 1d) and the intermittent WC-SC (Supplementary section SI5, the upper circuit in Fig. 1c) for some circuit motifs, as we explain in details in a later section.

## Results

### Oscillatory dynamics in one-gene and two-gene elements with time delay

**Figure 3:**
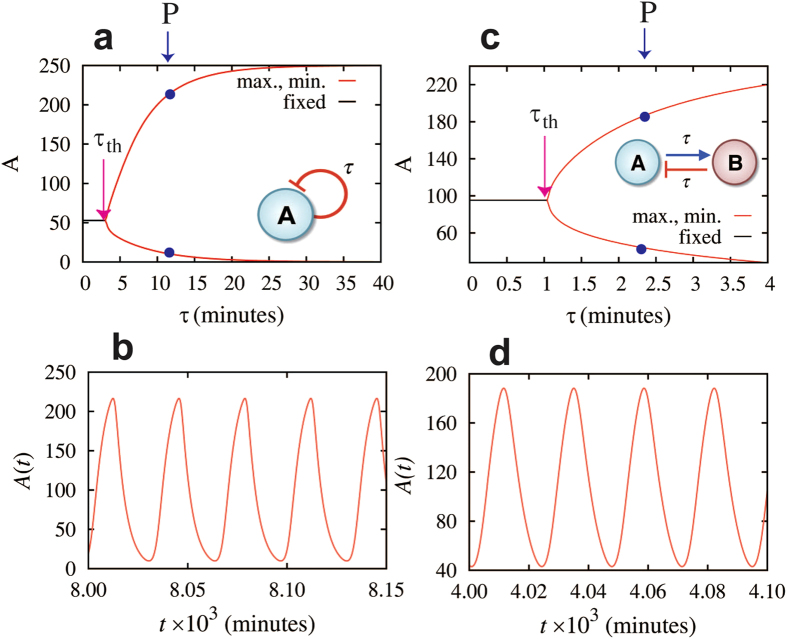
Oscillatory dynamics for elements with one delayed negative feedback loop. Left panels show the results for the element of the gene A with self-inhibition (inset of (**a**)); right panels are for the flip-flop element (two genes A and B with a forward activation and backward inhibition, inset of (**c**)). (**a**) and (**c**) show bifurcation diagrams of the fixed point (black line), the maximum level (upper red line), and minimum level (lower red line) of protein A with respect to different values of the time delay τ. When τ is less than the Hopf bifurcation point τ_th_ (3.1 minutes in (**a**) and 1.05 minutes in (**c**)), the circuits are in a steady state (black line for levels of protein A). When τ is larger than τ_th_, the circuit has dynamics of periodic oscillations. (**b**) and (**d**) show the time trajectories of the corresponding circuits illustrated in (**a**) and (**c**), where the time delays τ are set to τ = 12 minutes in (**b**) and τ = 2.4 minutes in (**d**) (blue points and arrows in (**a**) and (**c**)).

As is mentioned in the previous section, a self-inhibitory single gene element with time delay (Fig. 3a) has nearly the same dynamics as a classical repressilator composed of three identical genes (see Supplementary section SI1 for details). More specifically, such single-gene element exhibits a Hopf bifurcation, as function of the time delay, from a steady-state dynamics into an oscillatory one at a threshold delay 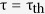 (Fig. 3a and 3b). The time dynamics of a self-inhibitory single-gene element with time delay τ is described by the following deterministic rate equation:


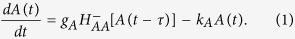


A represents the protein concentration measured in units of nM. Time t is measured in minutes. The protein production rate g_A_ is measured in units of nM/minute, and the degradation rate k_A_ is measured in units of 1/minute. These units are used throughout the article.

H^−^_AA_ is an inhibitory Hill function. In general, for gene X inhibited by gene Y, the inhibitory Hill function is defined by


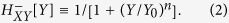


Where Y_0_ is the threshold concentration of the Hill function and n is its rank. Note that for self-inhibitory gene A discussed here, X≡Y≡A.

We note that the Hopf bifurcation from the steady-state (black curve in Fig. 3a) to the oscillatory dynamics (red curves that mark the maximum and minimum levels of A) occurs at time delay threshold τ_th_ = 3.1 minutes that is the order of 1/k_A_.

In Fig. 3c,d we demonstrate that a two-gene flip-flop element can give rise to oscillatory dynamics when time delay is included. This element also exhibits a Hopf bifurcation, as function of the time delay τ, from a steady-state dynamics into an oscillatory one at a threshold delay τ = τ_th_. The model equations for this two-gene motif are included in Supplementary section SI1. We note that the origin of the correspondence between the self-inhibitory one-gene element and the two-gene flip-flop element can be understood as follows. Since gene A activates its inhibitor gene B, it acts effectively as a self-inhibitory gene. Since each of the two regulatory paths (A-to-B and B-to-A) has a time delay τ, the flip-flop element corresponds to a single self-inhibitory gene with a time delay that is equal to twice τ plus a time delay associated with the dynamics of gene B. This is why the threshold time delay to induce oscillation (the bifurcation point) for the flip-flop element τ_th_ is 1.05 minutes, which is smaller than half of the time delay for the case of the single gene element.

### Weak chaotic dynamics in elements with two negative feedback loops

**Figure 4:**
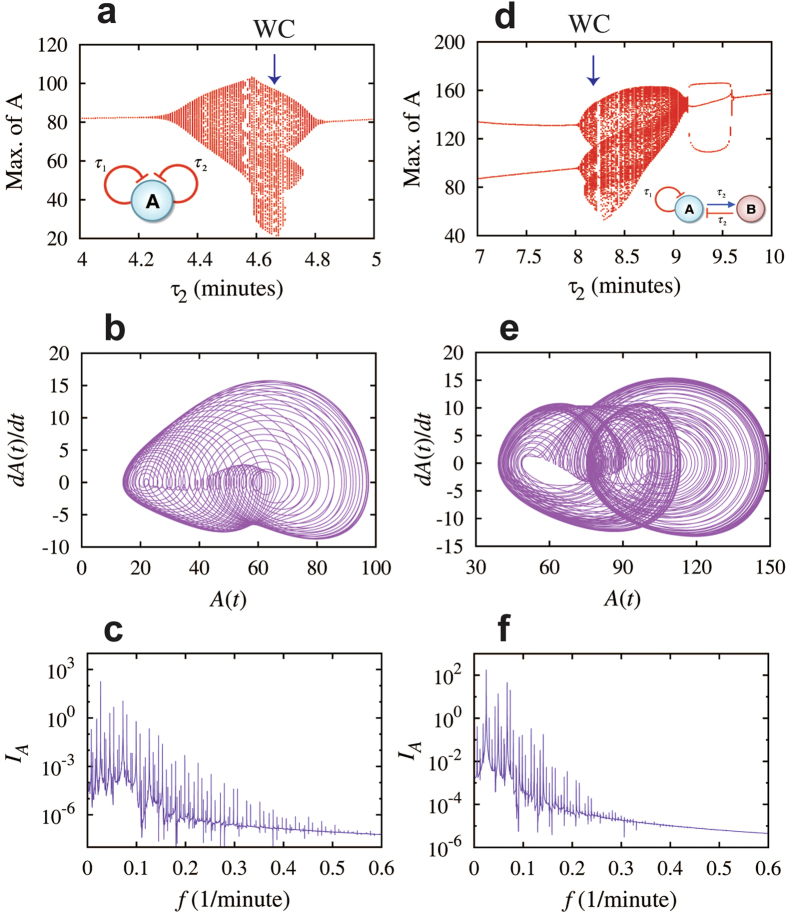
Weak chaotic dynamics for elements with two negative feedback loops. Left panels show the results for the element of the gene A with two delayed self-inhibitions (circuit diagram in inset of (**a**)). Panel (**a**) shows bifurcation of the maximum levels of protein A with respect to the values of time delay τ_2_ while fixing the time delay τ_1_ = 18 minutes. The dynamics is periodic when τ_2_ < 4.2 minutes or τ_2_ > 4.9 minutes, and quasi-periodic or weak chaotic dynamics otherwise. Panels (**b**) and (**c**) show the A(t) – dA(t)/dt maps and the full spectra of the protein level A for the case when τ_1_ = 18 minutes, τ_2_ = 4.65 minutes (navy arrow in (**a**)). Right panels show the results for the two-gene flip-flop element with time delays (circuit diagram in inset of (**d**)). Panels (**d**–**f**) are similar to panels (**a**–**c**). In the bifurcation diagram in (**d**), the time delay τ_1_ is fixed to be 5.3 minutes. The dynamics is periodic when τ_2_ < 8.0 minutes or τ_2_ > 9.18 minutes, and quasi-periodic or weak chaotic dynamics otherwise. Panels (**e**) and (**f**) are for the case when τ_1_ = 5.3 minutes, τ_2_ = 8.2 minutes (navy arrow in (**d**)). Circuits in both cases exhibit weak chaotic dynamics as illustrated by the maps (**b**,**e**), the full spectra (**c**,**f**) and the maximum-minimum spectra (see Supplementary section SI2).

In the previous section, we showed that a negative feedback loop, either in a single-gene or in a two-gene element, can give rise to periodic dynamics when there are time delays in the inhibitory regulation. Thus, it can be anticipated that circuit motifs with more than one negative feedback loops can exhibit more complex dynamics. Here, we investigate on two circuit motifs; one gene with two self-inhibitions (Fig. 4a) and a flip-flop element with one of the genes having a self-inhibition (Fig. 4d). Compared to the elements studied in the previous section (Fig. 3), each of the elements in this section contains an additional motif of time-delayed self-inhibition.

The time dynamics of a single gene element with two self-inhibitions with time delays τ_1_ and τ_2_, respectively, are described by the following deterministic rate equation:


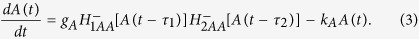


H^−^_1AA_ and H^−^_2AA_ are inhibitory Hill functions as defined by equation (2), representing the two self-inhibitions. Note that the time delays τ_1_ and τ_2_ are not necessarily the same. And in such a situation, the corresponding circuit can exhibit non-trivial dynamics.

To demonstrate this, we investigate the circuit dynamics while fixing the time delay for the first self-inhibition τ_1_ to be 18 minutes and varying the second time delay τ_2_. The rest circuit parameters are listed in Supplementary section SI2. In this specific case, the circuit dynamics always exhibit oscillations, regardless of the values of τ_2_. Thus, we plot a bifurcation diagram of the maximum levels of protein A for each oscillation with respect to the values of time delay τ_2_ (Fig. 4a). When τ_2_ varies from 4 minutes to 5 minutes, the circuit exhibits transitions from P to QP/WC dynamics, and back to P again. As an example, when τ_2_ = 4.65 minutes (navy arrow in Fig. 4a), the circuit exhibits a non-periodic oscillatory dynamics (Fig. 4b, Supplementary Fig. SI2.1). The corresponding full spectrum (Fig. 4c) and the maximum-minimum spectrum (Supplementary Fig. SI2.1) indicate this to be a weak chaotic dynamics (WC). We also found that WC/QP dynamics are observed when 3.09 minutes <τ_2_ < 3.113 minutes (Supplementary Fig. SI2.2).

Next we study the two-gene flip-flop element, where the first gene (A) has an additional self-inhibition (Fig. 4d). This two-gene element exhibits similar dynamics to the above-discussed one-gene element with two self-inhibitions. We considered a specific case in which gene A self-inhibition has time delay τ_1_, while the gene A activation of gene B and the backward inhibition of gene A by gene B have the same time delay τ_2_. One can also choose different time delays for the regulations in the flip-flop element, but similar dynamical behaviors will be observed. The model equations for this motif are included in Supplementary section SI2. For this circuit we considered the dynamics for τ_1_ equal to 5.3 minutes, and the rest parameters are listed in Supplementary section SI2. Similar to the previous circuit, the current motif also exhibits non-periodic dynamics when τ_2_ is approximately from 8 to 9.18 minutes. For example, when τ_2_ = 8.2 minutes (navy arrow in Fig. 4d), the circuit exhibits WC dynamics (Fig. 4e,f, Supplementary Fig. SI2.1).

### A possible mechanism to generate chaos for elements with two coupled feedback loops

In the previous two sections, we showed that elements with one negative feedback loop allow oscillations and elements with two coupled negative feedback loops can generate chaos. To understand the mechanism of the chaotic dynamics, we further study the one-gene element with two self-inhibitions (Fig. 4a). When τ_1_ = 18 minutes, the dynamics of the element have oscillations with a period of around 40 minutes for a large range of τ_2_ (Supplementary section SI2), including those of the P, QP, and WC cases mentioned above. In addition, when τ_1_ = 3 or 4 minutes, we also observed bifurcation of the dynamics from a steady-state one to an oscillatory one with respect to the value of τ_2_. The first bifurcation point τ_2_ ~ 3 minutes, which is very close to the range of τ_2_ to observe non-periodic dynamics of the same element (Fig. 4a and Supplementary Fig. SI2.2). This observation suggests that the first negative feedback loop drives the underlying oscillations and the second element switches frequently from the oscillatory phase to the stead-state phase, which probably causes the coupled motif to have quasi periodic or chaotic behavior. This mechanism could be generalized to circuit motifs with a positive feedback loop, which allows phase transitions between two stable steady states (next section).

### Strong chaotic dynamics in elements with one positive and two negative feedback loops

**Figure 5:**
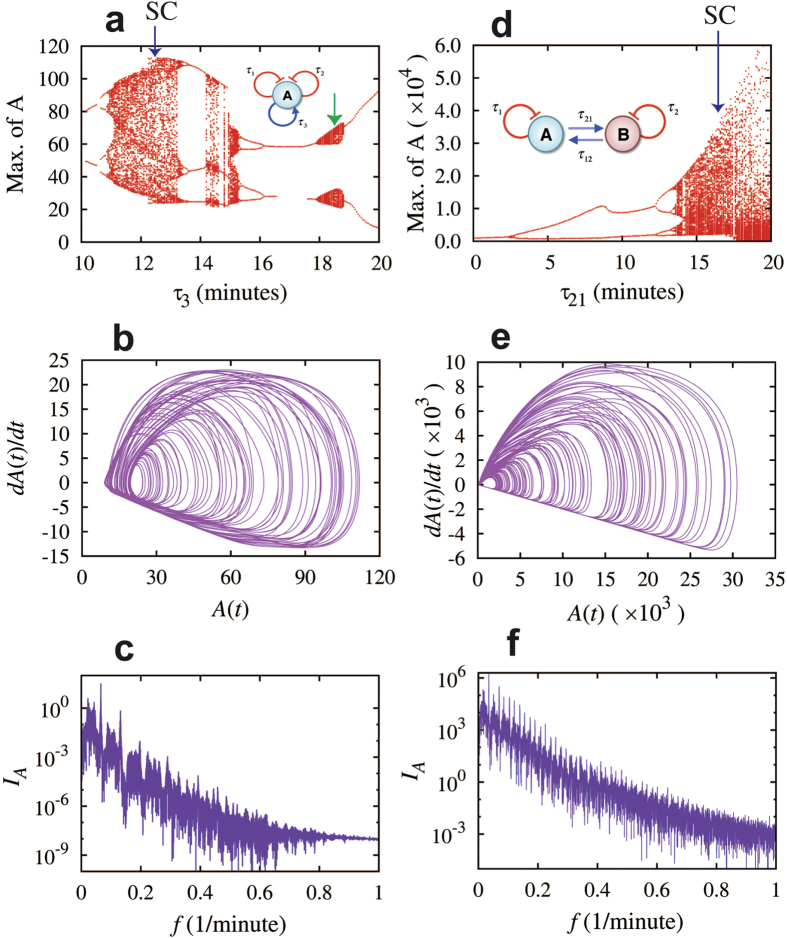
Strong chaotic dynamics for elements with two negative feedback loops and one positive loop. Left panels show the results for the element of the gene A with two delayed self-inhibitions and one delayed self-activation (circuit diagram in inset of (a)). Panel (**a**) shows bifurcation of the maximum levels of protein A with respect to the values of time delay τ_3_ while fixing the time delays τ_1_ = 18 minutes, τ_2_ = 8.0 minutes. The dynamics is non-periodic when τ_3_ is roughly in the range of 11 ~ 13, 14 ~ 15 and 18 ~ 19 minutes, and is periodic otherwise. Panels (**b**) and (**c**) show the A(t) – dA(t)/dt maps and the full spectra of the protein level A for the case when τ_3_ = 12.5 minutes (navy arrow in (a)). The dynamics for τ_3_ = 18.5 minutes (green arrow in (**a**)) is shown in the left column in Fig. 2 and Supplementary Fig. SI3. The quasi-periodic dynamics emerge in this case. Right panels show the results for the circuit of the two mutually activating and self-inhibitory genes A and B (circuit diagram in inset of (**d**)). Panels (**d**–**f**) are similar to panels (**a**–**c**). In the bifurcation diagram in (**d**) (maximum of A with respect to the time delay τ_21_), the time delay τ_1_ = 6.0 minutes, τ_2_ = 5.0 minutes, and τ_12_ = 7.5 minutes (navy arrow in (**d**)). The dynamics is non-periodic when τ_21_ is roughly larger than 14.0 minutes, and is periodic otherwise. Panels (**e**,**f**) are for the case when τ_21_ = 16.0 minutes. Circuits in both cases exhibit strong chaotic dynamics as illustrated by the maps (**b**,**e**), the full spectra (**c**,**f**) and the maximum-minimum spectra (see Supplementary section SI3).

In this section, we study a single-gene element with two self-inhibitions and one self-activation (inset of Fig. 5a), and a circuit with two self-inhibitory and mutually activating genes (Fig. 5d). Compared with those from the previous section, the gene elements here contain a positive feedback loop in addition to two negative feedback loops. We found that this combination of two negative feedback loops and one positive feedback loop can give rise to elaborate dynamics.

We first studied the single-gene element with three self-regulation loops with time delays τ_1_, τ_2_ and τ_3_, (Fig. 5a). The deterministic rate equation for this circuit is given by


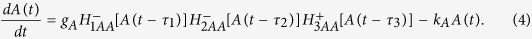


Here H^+^_3AA_ is an excitatory Hill function, representing the self-activation of gene A. By definition, H^+^_3AA_(A) ≡ 1-H^−^_3AA_(A).

We studied the dependence of the circuit dynamics on τ_3_ when setting τ_1_ to be equals to 18 minutes and τ_2_ to be equal to 8 minutes. The rest circuit parameters are listed in Supplementary section SI3. The corresponding bifurcation diagram is shown in Fig. 5a. By varying τ_3_ from roughly 10 to 20 minutes, we observed transitions among the P, QP, WC and SC dynamics. Interesting, the circuits exhibit non-periodic dynamics for a much wider range of time delays, compared to the circuits from the previous section. More detailed results are shown in Supplementary sections SI3 (QP, SC) and SI5 (WC).

Next we studied the two-gene circuit with two self-inhibitory and mutually activating genes (inset of Fig. 5d). Biologically, this circuit motif can correspond to single cell containing such circuit, or two positively interacting cells, each of which has a single-gene element with self-inhibition. Depending on the nature of the mutual activations, the circuit has slightly different non-periodic dynamics. If the mutual activations are modeled by excitatory Hill functions, the circuit can give rise to P, QP and WC dynamics (Supplementary section SI3). On the other hand, if the mutual activations are modeled by linear functions (corresponding to two interacting cells), the two cells can give rise to P, QP, WC, and SC dynamics (Fig. 5d–f, Supplementary Fig. SI3.3, and Fig. SI3.4).

We studied the dependence of the circuit dynamics on the time delay τ_21_ of the A-to-B activation when setting the first self-inhibition τ_1_ to be equal to 6.0 minutes, the second one τ_2_ to be equal to 5.0 minutes and the B-to-A activation time delay τ_12_ to be equal to 7.5 minutes where both activations are given by linear functions. The corresponding bifurcation diagram is shown in Fig. 5d. Similar to the single-gene element described above, this two-gene element can also give rise to elaborated non-periodic dynamics for a wide range of time delay τ_21_. More detailed results are shown in the Supplementary section SI3.

### Intermittency between periodic and strong chaotic mode in a circuit element with both self-inhibition and self-activation

**Figure 6:**
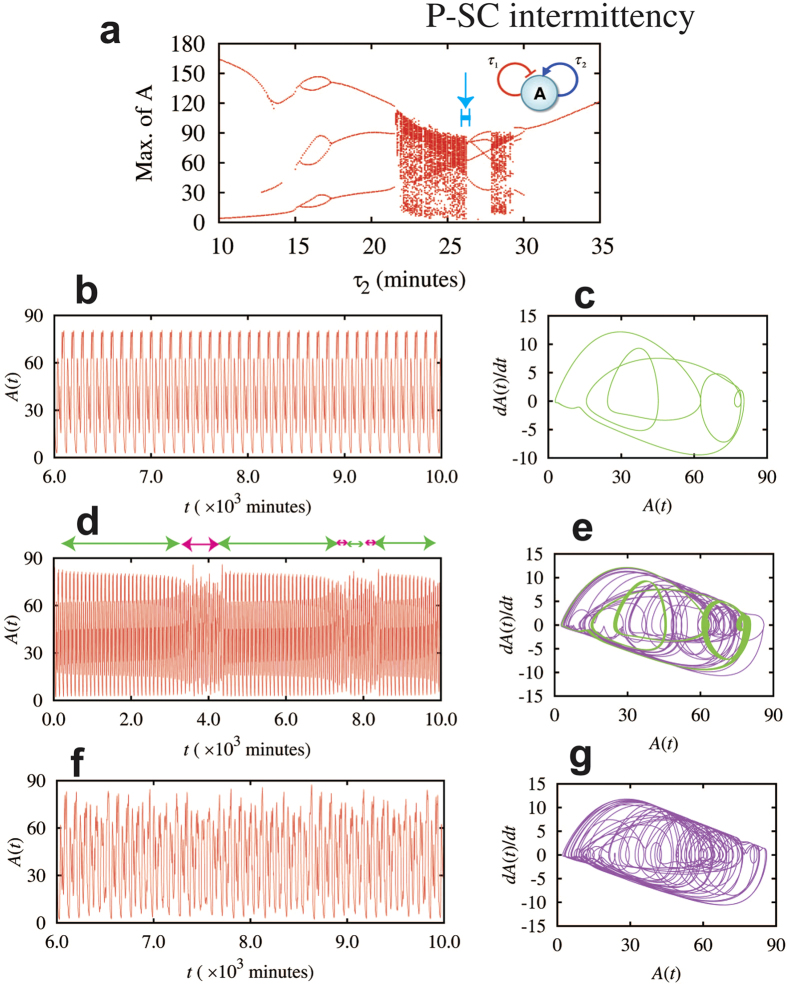
Intermittent dynamics between periodic and strong chaotic modes for a one-gene element with both self-inhibition and self-activation. The diagram of this element is shown in inset of panel (**a**). Panel (**a**) shows the bifurcation diagram of the maximum levels of protein A with respect to the time delay τ_2_ when τ_1_ is set to be 26.0 minutes. Here we calculated dynamics for cases in which τ_2_ values are selected to be slightly different, but all close to 26.0 minutes (the second row: τ_2_ = 26.3 minutes, the third row: τ_2_ = 26.29 minutes, the fourth row: τ_2_ = 26.0 minutes). A blue line with bars (pointed by a blue arrow) in panel (**a**) indicates the range of time delays in these cases. Panels (**b**), (**d**), and (**f**) show the time trajectories, and panels (**c**), (**e**), and (**g**) show the A(t) – dA(t)/dt maps. The green and purple lines in the phase-space map represent periodic and chaotic dynamics, respectively. When τ_2_ = 26.3 minutes (the second row), the circuit has periodic oscillatory dynamics; when τ_2_ = 26.0 minutes, the circuit has strong chaotic dynamics. Interestingly, when τ_2_ is in between (e.g. the third row), the circuit has intermittent dynamics between periodic and strong chaotic modes. In the time periods indicated by the green bidirectional arrows in (**d**), the circuit exhibits periodic oscillation with decayed amplitudes. The green line in (**e**) corresponds to the dynamics from 5.0 × 10^3^ to 6.5 × 10^3^ minutes in (**d**). The green line appears thick, because it consists of many oscillations with slightly different amplitudes. The magenta bidirectional arrows indicate time periods of strong chaos. The purple line in (**e**) corresponds to the time trajectory from 3.6 × 10^3^ to 4.4 × 10^3^ minutes, which is similar to the trajectory from a pure chaotic mode (panels (**f**), (**g**).

While the classification previously shown is a good characterization of the different dynamical regimes, some other circuits may show an even more complex dynamics that could not be either one of the dynamic regimes. During time evolution, they may switch among different dynamics, for example, in a single-gene element with both self-activation and self-inhibition (inset of Fig. 6a). The circuit is special in that it exhibits intermittency between periodic and chaotic behaviors in a single time trajectory.

Consider that the time delays for the self-inhibition and the self-activation are τ_1_ and τ_2,_ respectively, the deterministic rate equation for the circuit is


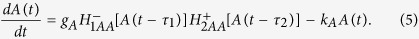


**Figure 7:**
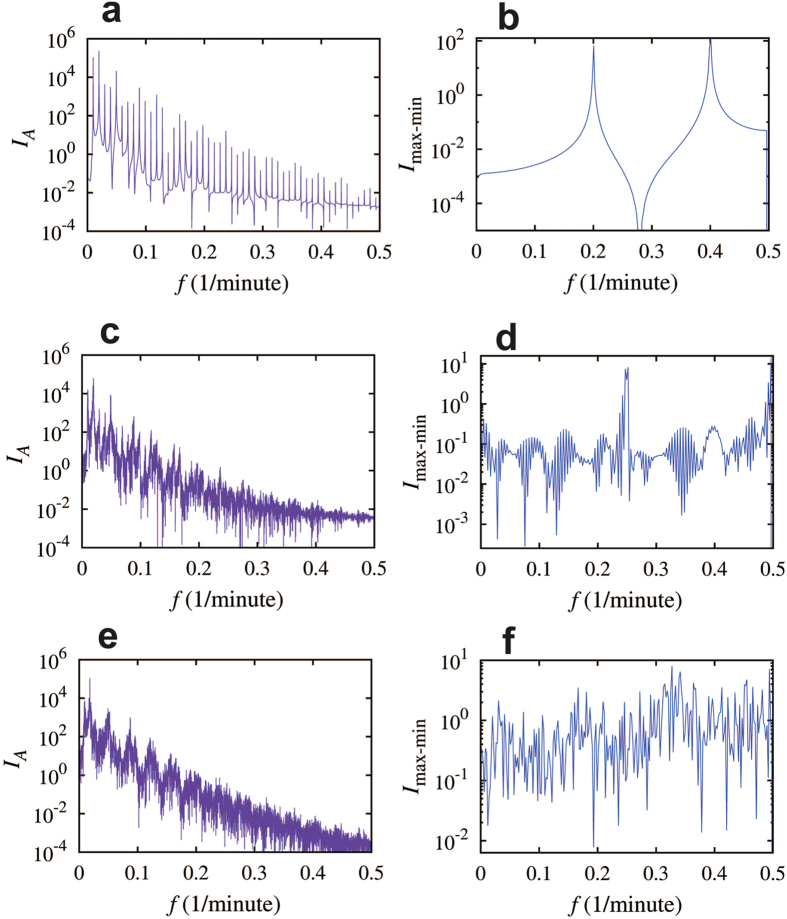
Full spectra and maximum-minimum spectra for the intermittent dynamics. The spectra correspond to the cases shown in Fig. 6. Left panels show the full spectra, and right panels show the maximum-minimum spectra. The first row corresponds to the case of pure periodic oscillatory dynamics (Fig.6b,c). There are discrete spikes in the full spectrum and only few spikes in the maximum- minimum spectrum. The third row corresponds to the case of pure chaotic dynamics (Fig. 6f,g). The second row corresponds to the case of the intermittency between periodic and strong chaotic mode (Fig. 6d,e). The full spectrum in panel (**c**) is similar to that in panel (**e**), because the chaotic component is dominant. On the other hand, the maximum-minimum spectra are very different for the two cases (panels (**d**) and (**f**)).

We studied the dependence of the circuit dynamics on the self-activation time delay τ_2_ when setting the self-inhibition time delay τ_1_ to be equal to 26 minutes. The rest circuit parameters are listed in Supplementary section SI4. The corresponding bifurcation diagram is shown in Fig. 6a. We found that this circuit exhibits periodic dynamics for majority of the τ_2_ values we have tested, but it has also non-periodic oscillations when τ_2_ varies between approximately 22 to 26 minutes, and 28 to 29 minutes. Close inspection of the dynamical behavior, especially for cases with τ_2_ that are close to the bifurcation point (e.g. around 26 minutes), we observed transitions from SC (τ_2_ = 26.0 minutes, Fig. 6f,g) to P (τ_2_ = 26.3 minutes, Fig. 6b,c) dynamics. Interestingly, for cases where the values of τ_2_ are in between, we observed intermittent dynamics between periodic and strong chaotic behaviors (i.e. P-SC intermittency, τ_2_ = 26.29 minutes, Fig. 6d,e). Here we refer the intermittent chaotic dynamics to the cases where substantially long durations of both periodic and chaotic dynamics are observed in a single trajectory, while transitions between these two modes are not periodic 43,47,48. We also noticed that, in the intermittent chaotic dynamics, the amplitudes of the oscillations in both modes decay slightly when time advances, until the mode is switched to the other one (Fig. 6d). Interestingly, the closer the τ_2_ to the bifurcation point around 26 minutes, the longer the time intervals of the periodic dynamics in each intermittent cycle, while the duration of the SC dynamics remains the same. Note that the full spectrum for the SC dynamics has mostly downward spikes (Fig. 7e), while that for the P dynamics has mostly upward and discrete spikes (Fig. 7a). As expected, that for intermittent dynamics has a mixture of both up and downward spikes (Fig. 7c). Moreover, the 2D map (A-dA/dt) for an intermittent dynamics (Fig. 6e) has both the trajectory of the periodic dynamics (green) and that of the chaotic dynamics (purple), indicating the coexistence of both modes. As shown in Supplementary section SI5, the one-gene element with two self-inhibitions and one self-activation (Fig. 5a) is another example of circuit that can generates intermittent dynamics, but in this case it is between the WC and the SC dynamics.

## Discussion

In this study, we showed that simple one-gene and two-gene elements can give rise to elaborate dynamics when time delays are included for the self and cross regulation loops. The observed time behaviors include periodic (P), quasi-periodic (QP), weak chaotic (WC) and strong chaotic (SC) dynamics as well as intermittent dynamics between periodic and chaotic behaviors. To quantitatively distinguish between the different dynamical modes, we developed a dedicated spectrum-based method that is essentially a combination of both the power-spectrum of the full dynamics and the power-spectrum of a series of discrete maximum/minimum expressions for each oscillation. Note that this combination of both spectra is needed to distinguish among the P, the QP, the WC and the SC dynamics. Either spectrum alone is insufficient to achieve this goal. We have also tried to describe the non-periodic dynamics by the Lyapunov exponents. However, the Lyapunov exponents obtained from a time series are sensitive to some parameters (e.g. embedding dimension and time lag for embedding), and it is hard to estimate these parameters for a time-delay nonlinear system^49^.

Our investigation reveals that, in order to generate chaotic dynamics, the circuit motifs need to have one negative feedback loop, which gives rise to oscillations, and another feedback loop (either positive, negative, or both) that creates two dynamical phases (e.g. two stable states, or a stable state and an oscillatory state). Chaos might take place when the first element generates sustainable oscillations while the second one allows hopping between the two dynamical modes. It is our expectation that this might be also true for several other chaotic motifs.

We also observed periodic/chaotic and weak/strong chaotic intermittency in certain circuit motifs. More efforts should be made to investigate the mechanism of the intermittent dynamics, in particular the dependence of the ratio between the time intervals of the two modes as function of the time delays. Another interesting future direction is to examine the effective landscape of the circuit 16,50,51,52,53 in the presence of time delays, and to obtain an understanding of the difference between the effective landscape for a chaotic system^54^ and a non-chaotic one.

Moreover, we demonstrated that the dynamics for certain circuit motifs can be converted from periodic oscillations to non-periodic, chaotic dynamics by just adjusting the values of time delays in a gene regulation. Note that time delays are common in gene-gene interactions. There are multiple mechanisms to adjust this time delays such as the effects of molecular crowding and the reduced efficacy of recruiting essential molecular components for gene regulation. Therefore, the delay-induced chaos could potentially affects normal cellular functions that typically require proper stable dynamics. Unlike some diseases which are caused by the malfunction of a gene, time delays in gene regulation provide an additional source for abnormal behavior.

The delay-induced chaos could also be relevant to diseases that are associated with cellular functions that require oscillatory gene expressions. For example, some cancer patients are known to suffer from an irregular circadian clock^55^,^56^, which in turn facilitates the advance of tumorigenesis by interfering with the immune system^57^. We hypothesize that cancer could induce additional time delays to the regulations of circadian genes, therefore generating non-periodic dynamics which disrupt the usual process. Indeed, the core circadian gene regulatory circuit could potentially have delay-induced chaos because the circuit motif has a negative feedback loop and a positive feedback loop (Fig. 1e)^58^. Chaotic behavior has also recently been studied in the context of interactions between cancer and the immune system^59^. A good example is the NFκB genetic circuit, which plays important roles in both immune response and proliferation signaling. Again, the NFκB circuit (Fig.1f) is composed of two coupled negative feedback loops^60^. The normal functions of NFκB could be potentially affected by non-periodic expression dynamics of the circuit. Since time delays are inherent in every gene network, it is our expectation that several additional biological processes will be affected by delay induced chaos.

## Additional Information

**How to cite this article**: Suzuki, Y. *et al.* Periodic, Quasi-periodic and Chaotic Dynamics in Simple Gene Elements with Time Delays. *Sci. Rep.* **6**, 21037; doi: 10.1038/srep21037 (2016).

## Supplementary Material

Supplementary Information
