# Supplementary material for: Periodic, Quasi-periodic and Chaotic Dynamics in Simple Gene Elements with Time Delays
Source: Sci Rep. 2016 Feb 15;6:21037. doi: 10.1038/srep21037 (PMC4753448; doi:10.1038/srep21037)
Supplement: Supplementary Information [file srep21037-s1.pdf]

Periodic, Quasi-periodic and Chaotic Dynamics in Simple Gene Elements with Time Delays

Yoko Suzuki, Mingyang Lu, Eshel Ben-Jacob, José N. Onuchic

Supplementary Information

SI 1 Elements with one negative feedback

SI1.1 Models and parameters (Fig. 3)

In this section, we provide the details for modeling elements with one negative feedback loop in Fig. 3. Model parameters of the self-inhibitory single gene element with time delay are given in Table SI1.1.

Table SI1.1: **The parameters for the self-inhibitory single gene element with time delay model (Eqs. (1) and (2)) in the main article.**

Parameter	Value
g_A	50 (nM/minute)
k_A	0.20 (minute^{-1})
A_0	38 (nM)
n	4

The time trajectory of the two-gene flip-flop element with time delay τ (the lower circuit in Fig. 1(a)) is described by

$$\frac{dA(t)}{dt} = g_A H^{-}_{AB}[B(t-\tau)] - k_A A(t) \quad \text{(SI1.1)}$$

$$\frac{dB(t)}{dt} = g_B H^{-}_{BA}[A(t-\tau)] - k_A B(t) \quad \text{(SI1.2)}$$

Model parameters in Eqs. (SI1.1) and (SI1.2) are given in Table. SI1.2.

SI1.2 Oscillatory dynamics of the elements

In Fig. 3 in the main article, we have shown the bifurcation and time dependence for the self-inhibitory single gene element with time delay and the two-gene flip-flop element

Table SI1.2: **The parameters for the two-gene flip-flop element with time delay (Eqs. (SI1.1) and (SI1.2)).**

Parameter	Value
g_A	50 (nM/minute)
g_B	50 (nM/minute)
k_A	0.20 (minute^{-1})
k_B	0.20 (minute^{-1})
$A_{0,BA}$	100 (nM)
$B_{0,AB}$	100 (nM)
n_{AB}	4
n_{BA}	4

with time delay. In order to compare oscillatory dynamics with quasi-periodic, weak-chaotic, and strong-chaotic dynamics, here we show the map and the power spectrum of the oscillatory dynamics.

Figure SI1.1 shows the results for the self-inhibitory single gene element (left panels) and those for the two-gene flip-flop element (right panels). (a) and (d) show phase-space maps $A(t)$ - $dA(t)/dt$; (c) and (f) show the power spectra, which is similar to those of the quasi-periodic and weak-chaotic dynamics; (b) and (e) show another phase-space maps $A(t-T)$ - $A(t)$, where T is a period of oscillatory motion. The results suggest that the phase-space maps are good in identifying the oscillatory dynamics from the other dynamics.

SI1.3 A reduced model of the classical repressilator

In this section, we show the classical repressilator, a gene circuit with three components (Fig. SI1.2(a)), can be reduced to a self-inhibitory one-gene element with time delay (Fig. SI1.2(b)) as mentioned in the introduction in the main article.

The deterministic equations of a classical ABC repressilator are described by

$$\begin{aligned}\frac{dA(t)}{dt} &= g_A H^{-}_{AC}[C(t)] - k_A A(t),\\ \frac{dB(t)}{dt} &= g_B H^{-}_{BA}[A(t)] - k_B B(t),\\ \frac{dC(t)}{dt} &= g_C H^{-}_{CB}[B(t)] - k_C C(t),\end{aligned} \tag{SI1.3}$$

where g_X and k_X (X stands for A, B, and C) are synthesis and degradation rates, respectively. $H^{-}_{AC}(C)$, $H^{-}_{BA}(A)$, and $H^{-}_{CB}(B)$ are inhibitory Hill functions defined by Eq. (2). The rank and the mid point concentration of the Hill function $H^{-}_{XY}(Y)$ are given by n_{XY} and Y_0, respectively. For simplicity, we set $g_A = g_B = g_C = g$, $k_A = k_B = k_C = k$, and $n_{AC} = n_{BA} = n_{CB} = n$, $A_0 = B_0 = C_0$. In this case, the inhibitory Hill functions become identical, i.e., $H^{-}_{AC}(X) = H^{-}_{BA}(X) = H^{-}_{CB}(X) = H^{-}(X)$. We call these parameter sets as symmetric parameter sets.

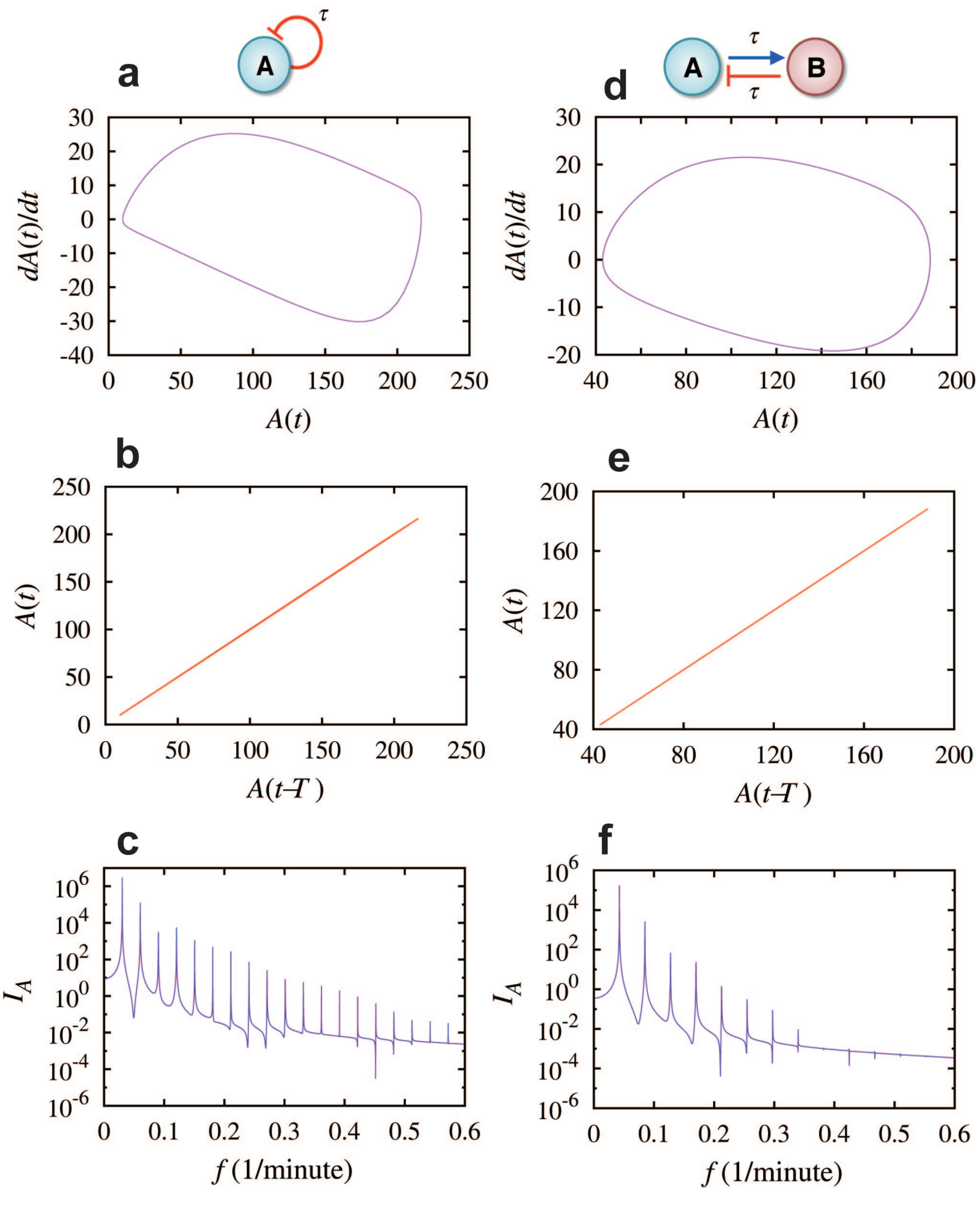

Figure SI1.1: **Oscillatory dynamics in the one and two- gene elements with time delay.** Left panels show the results for the circuit of the gene A with a time-delayed self-inhibition (circuit diagram above panel (a)); right panels for the two-gene (A and B) flip-flop element with time delay (circuit diagram above panel (d)). Same as Fig. 3, the time delay τ is 12(minutes) in the left panels and 2.4(minutes) in the right panels. (a) and (d) show phase-space maps $A(t)$ - $dA(t)/dt$, illustrating that the dynamics in both cases reach to a stable limit cycle. (b) and (e) show another phase-space maps $A(t-T)$ - $A(t)$, where T is the period of the oscillation. It shows that $A(t)$ always equals to $A(t-T)$, indicating a periodically oscillatory dynamics. (c) and (f) show the power spectra of $A(t)$.

For some parameters, the time trajectory of the classical repressilator converge to limit cycles. This oscillatory motions can be reproduced by self-inhibitory one-gene element with time-delay. We show an example in Fig. SI1.2(c)-(e), where the model parameters are given in Table SI1.3.

Table SI1.3: **The parameters for the classical repressilator (Eqs. (SI1.3)).**

Parameter	Value
g_A, g_B, g_C	50 (nM/minute)
k_A, k_B, k_C	0.20 (minute^{-1})
A_0, B_0, C_0	100 (nM)
n_{AC}, n_{BA}, n_{CB}	4

Next, we analytically explain the equivalence between the the two motifs. Eq. (SI1.3) are rewritten as

$$\frac{d}{dt}\begin{pmatrix} A(t)-A_{\mathrm{f}} \\ B(t)-B_{\mathrm{f}} \\ C(t)-C_{\mathrm{f}} \end{pmatrix} = \begin{pmatrix} g(H(C(t))-H(C_{\mathrm{f}}))-k(A(t)-A_{\mathrm{f}}) \\ g(H(A(t))-H(A_{\mathrm{f}}))-k(B(t)-B_{\mathrm{f}}) \\ g(H(B(t))-H(B_{\mathrm{f}}))-k(C(t)-C_{\mathrm{f}}) \end{pmatrix}$$
$$= \begin{pmatrix} -k & 0 & h \\ h & -k & 0 \\ 0 & h & -k \end{pmatrix}\begin{pmatrix} A(t)-A_{\mathrm{f}} \\ B(t)-B_{\mathrm{f}} \\ C(t)-C_{\mathrm{f}} \end{pmatrix} + \sum_{n=2}^{\infty}\frac{1}{n!}\begin{pmatrix} H^{-\,(n)}(C_{\mathrm{f}})(C(t)-C_{\mathrm{f}})^n \\ H^{-\,(n)}(A_{\mathrm{f}})(A(t)-A_{\mathrm{f}})^n \\ H^{-\,(n)}(B_{\mathrm{f}})(B(t)-B_{\mathrm{f}})^n \end{pmatrix}, \tag{SI1.4}$$

where X_{f} $(X = A, B, C)$ is a fixed point of X satisfying $gH^{-}(gH^{-}(gH^{-}(X))) = X$. h is given by $h = gH^{-\,(1)}(X_{\mathrm{f}})$, where $H^{-\,(n)}(X) \equiv d^nH^{-}(X)/dX^n$ $(n = 1, 2, \ldots)$. The eigenvalues of a matrix F,

$$F \equiv \begin{pmatrix} -k & 0 & h \\ h & -k & 0 \\ 0 & h & -k \end{pmatrix}, \tag{SI1.5}$$

are given by $\lambda_1 = -k + h(< 0)$, $\lambda_2 = -k + he^{\mathrm{i}\varphi}$, $\lambda_3 = -k + he^{-\mathrm{i}\varphi}$, where $\varphi = 2\pi/3$. Hence $k = -h/2$ is the Hopf bifurcation point, because the real parts of λ_2 and λ_3 are zero. The levels of proteins A, B, and C converge to limit cycles under the condition $k \geq -h/2$.

For the symmetric case, the levels of proteins A, B, and C satisfy the following relation after the convergence to the limit cycle with same period T,

$$B(t) = A(t + \frac{T}{3}) = A(t - \frac{2T}{3}),\ \ C(t) = A(t + \frac{2T}{3}) = A(t - \frac{T}{3}). \tag{SI1.6}$$

The relation can be derived by using the following argument.

We assume that $B(t)$ and $C(t)$ are given by $B(t) = A(t - \alpha)$ and $C(t) = A(t - \beta)$ for

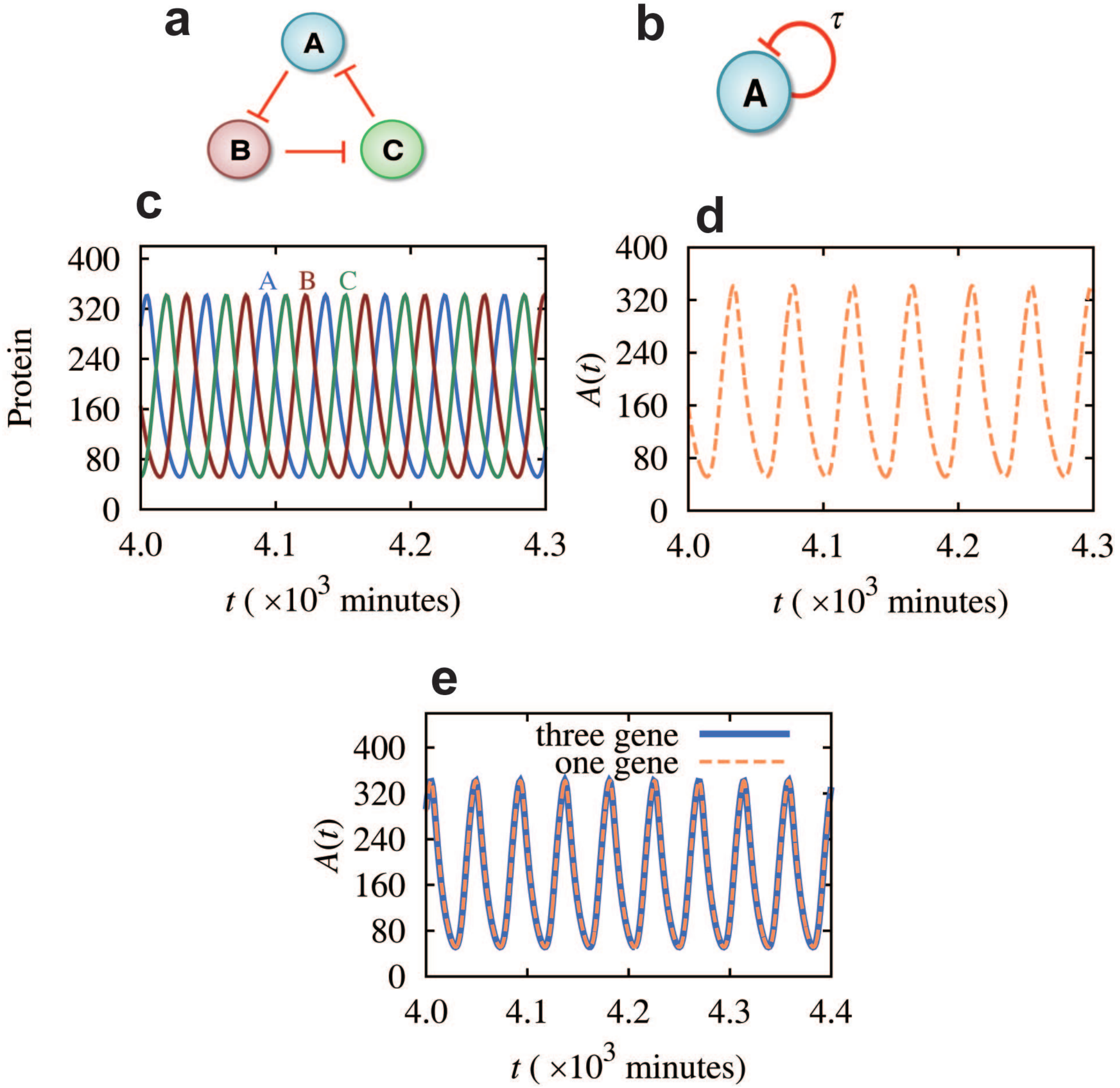

Figure SI1.2: **Comparison of the classical repressilator with the self-inhibitory one-gene element with time-delay.** (a) is the circuit diagram of the classical repressilator and (b) is the circuit of self-inhibitory one-gene element with time-delay. (c) and (d) show the time trajectory of the circuits in (a) and (b) respectively. In (c), the levels of protein A (blue), B (red), and C (green) oscillate with a period $T = 44.15$ (minutes) for the protein production rates $g_A = g_B = g_C = 50$ (nM/minute) and the degradation rates $k_A = k_B = k_C = 0.1$ (minute^{-1}). In (d), the protein level A oscillates with the same vales of the protein production rate and the degradation rate as (c); $g_A = 50$ (nM/minute) and $k_A = 0.1$ (minute^{-1}), where the time delay is set to $\tau = T/3$. (e) shows that the level of protein A in (c) (blue solid line) is identical to that of protein A in (d) (red dashed line) after a time shift of -15.4 (minutes) (red dashed line in (e)).

large t. Eq. (SI1.3) are written as

$$\frac{dA(t)}{dt} = f[A(t-\beta)] - kA(t), \tag{SI1.7}$$

$$\frac{dA(t-\alpha)}{dt} = f[A(t)] - kA(t-\alpha), \tag{SI1.8}$$

$$\frac{dA(t-\beta)}{dt} = f[A(t-\alpha)] - kA(t-\beta). \tag{SI1.9}$$

Eqs. (SI1.8) and (SI1.9) are written as

$$\frac{dA(t)}{dt} = f[A(t+\alpha)] - kA(t), \tag{SI1.10}$$

$$\frac{dA(t)}{dt} = f[A(t+\beta-\alpha)] - kA(t). \tag{SI1.11}$$

By comparing Eqs. (SI1.7), (SI1.10), and (SI1.11), we obtain

$$-\beta = \alpha + m_2 T, \tag{SI1.12}$$

$$\alpha + m_2 T = \beta - \alpha + m_1 T, \tag{SI1.13}$$

$$\beta - \alpha + m_1 T = -\beta, \tag{SI1.14}$$

where m_1 and m_2 are integers. From Eqs. (SI1.12), (SI1.13), and (SI1.14),

$$\alpha = \frac{m_1 - 2m_2}{3}\,T,\ \beta = \frac{-m_1 - m_2}{3}\,T. \tag{SI1.15}$$

The relations (SI1.6) are reproduced for $m_1 = m_2 = -1$. The other solution is

$$B(t) = A(t - \frac{T}{3}) = A(t + \frac{2T}{3}),\ C(t) = A(t - \frac{2T}{3}) = A(t + \frac{T}{3}), \tag{SI1.16}$$

for $m_1 = m_2 = 1$. One of the sets is for the current motif but the other is none.

In order to identify m_1 and m_2, we study Eq. (SI1.4) around the Hopf bifurcation point, $k = -h/2$;

$$\frac{d}{dt}\mathbf{z}(t) = F_{\mathrm{h}}\mathbf{z}(t) + \mathbf{g}(\mathbf{z}(t)), \tag{SI1.17}$$

where

$$F_{\mathrm{h}} = \begin{pmatrix} -k & 0 & -2k \\ -2k & -k & 0 \\ 0 & -2k & -k \end{pmatrix}, \tag{SI1.18}$$

$$\mathbf{z}(t) = \begin{pmatrix} A(t) - A_{\mathrm{f}} \\ B(t) - B_{\mathrm{f}} \\ C(t) - C_{\mathrm{f}} \end{pmatrix}, \tag{SI1.19}$$

$$\mathbf{g}(\mathbf{z}(t)) = (h+2k)\begin{pmatrix} z_3(t) \\ z_1(t) \\ z_2(t) \end{pmatrix} + \sum_{n=2}^{\infty} \frac{h_n}{n!} \begin{pmatrix} [z_3(t)]^n \\ [z_1(t)]^n \\ [z_2(t)]^n \end{pmatrix}, \tag{SI1.20}$$

$$h_n = g\left(\frac{d^n H^-(A)}{dA^n}\right)_{A=A_{\mathrm{f}}}. \tag{SI1.21}$$

From Eq. (SI1.17), $\mathbf{z}(t)$ is written as

$$\mathbf{z}(t) = \mathrm{e}^{F_\mathrm{h} t}\,\mathbf{z}(0) + \mathrm{e}^{F_\mathrm{h} t}\int_0^t dt'\ \mathrm{e}^{-F_\mathrm{h} t'}\mathbf{g}(\mathbf{z}(t')). \tag{SI1.22}$$

By using

$$F_\mathrm{h} = U^\dagger \Lambda(t) U, \tag{SI1.23}$$

$$\Lambda = \begin{pmatrix} \mathrm{e}^{\lambda_1 t} & 0 & 0 \\ 0 & \mathrm{e}^{\lambda_2 t} & 0 \\ 0 & 0 & \mathrm{e}^{\lambda_3 t} \end{pmatrix}, \tag{SI1.24}$$

$$U = \frac{1}{\sqrt{3}}\begin{pmatrix} 1 & 1 & 1 \\ 1 & \mathrm{e}^{\mathrm{i}\varphi} & \mathrm{e}^{-\mathrm{i}\varphi} \\ 1 & \mathrm{e}^{-\mathrm{i}\varphi} & \mathrm{e}^{\mathrm{i}\varphi} \end{pmatrix}, \tag{SI1.25}$$

where $U^\dagger U = U U^\dagger = 1$, we obtain

$$\mathrm{e}^{F_\mathrm{h} t} = \frac{\mathrm{e}^{-\nu t}}{3}\begin{pmatrix} 1 & 1 & 1 \\ 1 & 1 & 1 \\ 1 & 1 & 1 \end{pmatrix} + \frac{2\mathrm{e}^{-\mu t}}{3}\begin{pmatrix} \cos(\omega t) & \cos(\omega t - \varphi) & \cos(\omega t + \varphi) \\ \cos(\omega t + \varphi) & \cos(\omega t) & \cos(\omega t - \varphi) \\ \cos(\omega t - \varphi) & \cos(\omega t + \varphi) & \cos(\omega t) \end{pmatrix}. \tag{SI1.26}$$

Here $\nu \equiv -\lambda_1 = 3k$, $\mu = -\mathrm{Re}\ \lambda_2 = -\mathrm{Re}\ \lambda_3 = 0$, and $\omega \equiv -\mathrm{Im}\ \lambda_2 = \mathrm{Im}\ \lambda_3 = \sqrt{3}k$. For large t, the first term of Eq. (SI1.22) is given by

$$\mathrm{e}^{F_\mathrm{h} t}\mathbf{z}(0) = \frac{2}{3}\begin{pmatrix} z_1(0)\cos(\omega t) + z_2(0)\cos[\omega(t-\frac{T}{3})] + z_3(0)\cos[\omega(t+\frac{T}{3})] \\ z_1(0)\cos[\omega(t+\frac{T}{3})] + z_2(0)\cos(\omega t) + z_3(0)\cos[\omega(t-\frac{T}{3})] \\ z_1(0)\cos[\omega(t-\frac{T}{3})] + z_2(0)\cos[\omega(t+\frac{T}{3})] + z_3(0)\cos(\omega t) \end{pmatrix}, \tag{SI1.27}$$

where $T = 2\pi/\omega$. Therefore the first term of Eq. (SI1.22) satisfies the relations Eq. (SI1.6). In this case, as we discussed using Eq. (SI1.15), $m_1 = m_2 = -1$.

By substituting Eq. (SI1.6) into Eq. (SI1.3), the three equations are reduced to the following relation,

$$\frac{dA(t)}{dt} = gH^-(A(t-\frac{T}{3})) - kA(t). \tag{SI1.28}$$

SI 2 Elements with two negative feedbacks

SI2.1 Models and parameters (Fig. 4)

In this section, we show the details for modeling elements with two negative feedback loops (Fig. 4). Table SI2.1 shows the model parameters of the single gene element with two self-inhibitions, each of which has different time delays, τ_1 and τ_2 ($\tau_1 \neq \tau_2$).

Table SI2.1: **Model parameters for the single gene element with two self-inhibitions (Eq. (3)) in the main article.**

Parameter	Value
g_A	50 (nM/minute)
k_A	0.20 (minute^{-1})
$A_{0,1AA}$	38 (nM)
$A_{0,2AA}$	38 (nM)
n_{1AA}	4
n_{2AA}	2

The time trajectory of two-gene flip-flop element with one-sided self-inhibition (the inset of Fig. 4d in the main article) is described by

$$\frac{dA(t)}{dt} = g_A H^-_{AA}[A(t-\tau_1)] H^-_{AB}[B(t-\tau_2)] - k_A A(t) \quad \text{(SI2.1)}$$

$$\frac{dB(t)}{dt} = g_B H^-_{BA}[A(t-\tau_2)] - k_A B(t) \quad \text{(SI2.2)}$$

Model parameters in Eqs. (SI2.1) and (SI2.2) are given in Table SI2.2.

Table SI2.2: **Model parameters for the two-gene flip-flop element with one-sided self-inhibition described by Eqs. (SI2.1) and (SI2.2).**

Parameter	Value
g_A	50 (nM/minute)
g_B	50 (nM/minute)
k_A	0.20 (minute^{-1})
k_B	0.20 (minute^{-1})
$A_{0,AA}$	100 (nM)
$B_{0,AB}$	100 (nM)
$A_{0,BA}$	100 (nM)
n_{AA}	4
n_{AB}	4
n_{BA}	4

SI2.2 Weak chaotic and quasi-periodic dynamics of the elements

In Fig. 4 in the main article, we have shown that the above-mentioned elements are capable of generating weak chaotic dynamics, from the analysis of the bifurcation of the maximum levels with respect to the values of the time delays, the 2D map of time trajectory in the phase space of $A(t)$ - $dA(t)/dt$, and the full spectrum. We here show additional features of the dynamics, including the time trajectories, the 2D map of $A(t - \tau_0)$ - $A(t)$, and the maximum-minimum spectrum.

Figure SI2.1 shows the results for the single gene element with two self-inhibitions (left panels) and those for the two-gene flip-flop element where the first gene A has an additional self-inhibition (right panels). (a) and (d) show the time trajectories. (b) and (e) show the phase-space maps of $A(t - \tau_0)$ - $A(t)$, where τ_0 is set to be 37 minutes for (b) and is set to be 41 minutes for (e). Note the value of τ_0 is chosen to be $1/f_0$, where f_0 is the frequency of the maximum point of the full spectra (Figs 4(c) and (f) in the main article). (c) and (f) show the maximum-minimum spectra.

From the results of both Fig. 4 in the main article and Fig. SI2.1, we conclude that the circuits shown in the insets of Figs. 4(a) and (d) can have weak chaotic dynamics.

In addition to the weak chaotic dynamics, the same elements also allow quasi-periodic dynamics. The model parameters are the same as shown in Table SI2.1, except for different values of time delays. We show the zoomed-in bifurcation diagram of the maximum levels of protein A with respect to the values of the time delay τ_2 when the time delay τ_1 is set to be 18 minutes (Figs. SI2.2). We found that the circuit exhibits non-periodic oscillatory dynamics when 3.09 minutes $< \tau_2 <$ 3.115 minutes (Fig. SI2.2(b)) and 4.3 minutes $< \tau_2$ $<$ 4.8 minutes (Fig. SI2.2(c)). For $\tau_2 = 3.113$ minutes (green arrow in Fig. SI2.2(b)), the circuit exhibits a weak chaotic dynamics as shown in Fig. SI2.3. For $\tau_2 = 4.4$ minutes (green arrow in Fig. SI2.2(c)), the circuit exhibits a quasi-periodic dynamics as shown in Fig. SI2.4.

Similarly, the two-gene flip-flop element with one-sided self-inhibition also exhibits quasi-periodic dynamics, are shown in Figs. SI2.5 and SI2.6. Figure SI2.5 shows the zoomed-in bifurcation of the maximum levels of protein A with respect to the values of the time delay τ_2 when the time delay τ_1 is set to be 5.3 minutes. We found that the circuit exhibits non-periodic oscillatory dynamics when 8.0 minutes $< \tau_2 <$ 9.1 minutes (Fig. SI2.5(b)) and 16.4 minutes $< \tau_2 <$ 18.8 minutes (Fig. SI2.5(c)). For $\tau_2 = 8.05$ minutes (green arrow in Fig. SI2.5(b)), the circuit exhibits quasi-periodic dynamics as shown in Fig. SI2.6. For $\tau_2 = 16.8$ minutes (green arrow in Fig. SI2.5(c)), the circuit exhibits a weak chaotic dynamics as shown in Fig. SI2.7.

SI2.3 Coupling of two negative feedback loops

In the main article, we showed that the element exhibits weak chaos when $\tau_1 = 18$ minutes and $\tau_2 \simeq 3$ minutes. In this section, we study the coupling of the two negative feedback loops in the one-gene element with two delayed self-inhibitions.

We first evaluate the circuit dynamics for $\tau_1 = 18$ minutes. When there is no time delay in the second negative feedback loop, i.e., $\tau_2 = 0.0$ minutes, the circuit has oscillatory dynamics with a period of about 40 minutes (Fig. SI2.8(a)). When $\tau_2 = 4.0$ minutes,

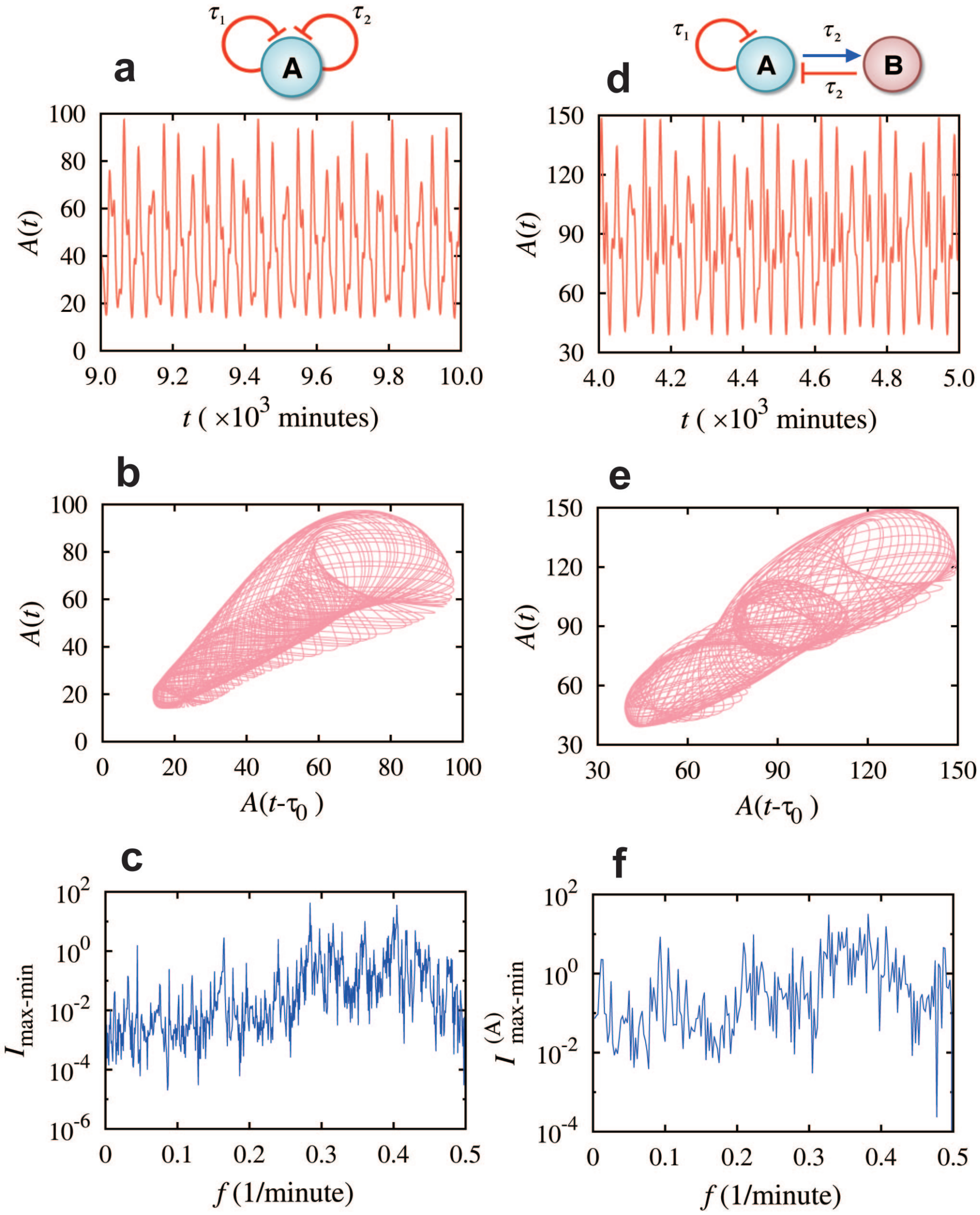

Figure SI2.1: **Weak chaotic dynamics in the circuit elements with two negative feedback loops.** Left panels show the results for the circuit of the gene A with two delayed self-inhibitions (circuit diagrams above panel (a)); right panels for the two-gene (A and B) flip-flop element with time delays (circuit diagram above panel (d)) where the gene A has an additional self-inhibition. Same as Fig. 4 in the main article, the time delays are set to $\tau_1 = 18$ minutes and $\tau_2 = 4.65$ minutes for the left panels and $\tau_1 = 5.3$ minutes and $\tau_2 = 8.2$ minutes in the right panels. (a) and (d) show the time trajectory. (b) and (e) show the phase-space maps $A(t - \tau_0)$ - $A(t)$, where τ_0 is set to be 37 minutes in (b) and is set to 41 minutes in (e). The time lag τ_0 corresponds to the frequency of the maximum point of the full spectrum (Figs. 4(c) and (f) in the main article). (c) and (f) show the maximum-minimum spectra of $A(t)$.

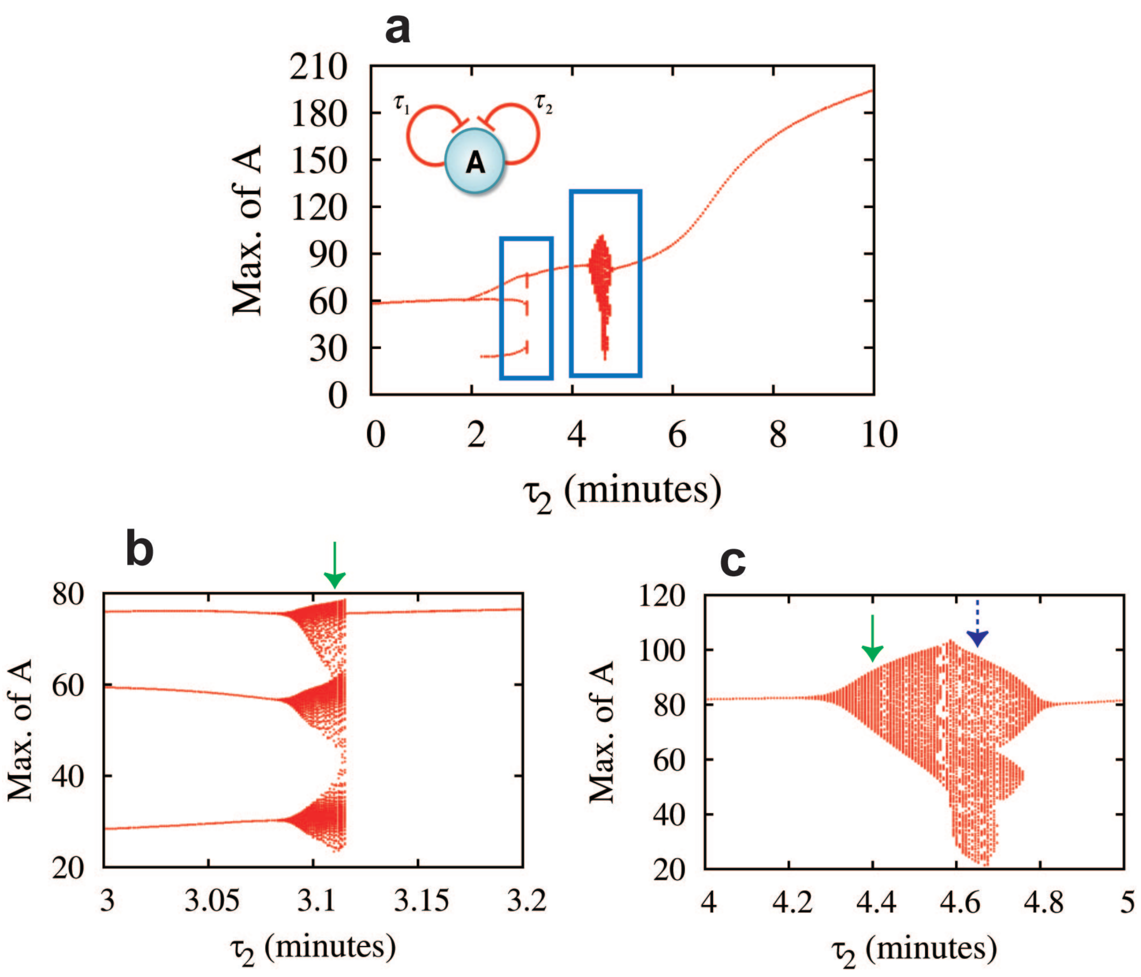

Figure SI2.2: **Bifurcation diagrams of the maximum levels of A for the one gene element with two self-inhibitions.** Panel (a) shows the bifurcation of the maximum levels of protein A (circuit diagram in the inset of panel (a)) with respect to the values of the time delay τ_2 when the time delay τ_1 is set to be 18 minutes. In the bifurcation, non-periodic dynamics are shown around $\tau_2 = 3.0$ minutes (left blue square in panel (a)) and around $\tau_2 = 4.5$ minutes (right blue square in panel (a)). The detailed bifurcations for these regions are shown in panels (b) and (c). The dynamics is non-periodic for 3.09 minutes $< \tau_2 <$ 3.115 minutes (panel (b)) and 4.3 minutes $< \tau_2 <$ 4.8 minute (panel (c)). The circuit exhibits weak chaotic dynamics for $\tau_2 = 3.113$ minutes (green arrow in panel (b)) as in Fig. SI2.3 and for $\tau_2 = 4.65$ minutes (blue arrow in panel (c)) as in Fig. 4 in the main article and Fig. SI2.1. The circuit exhibits quasi-periodic dynamics for $\tau_2 = 4.4$ minutes (green arrow in panel (c)) as in Fig. SI2.4.

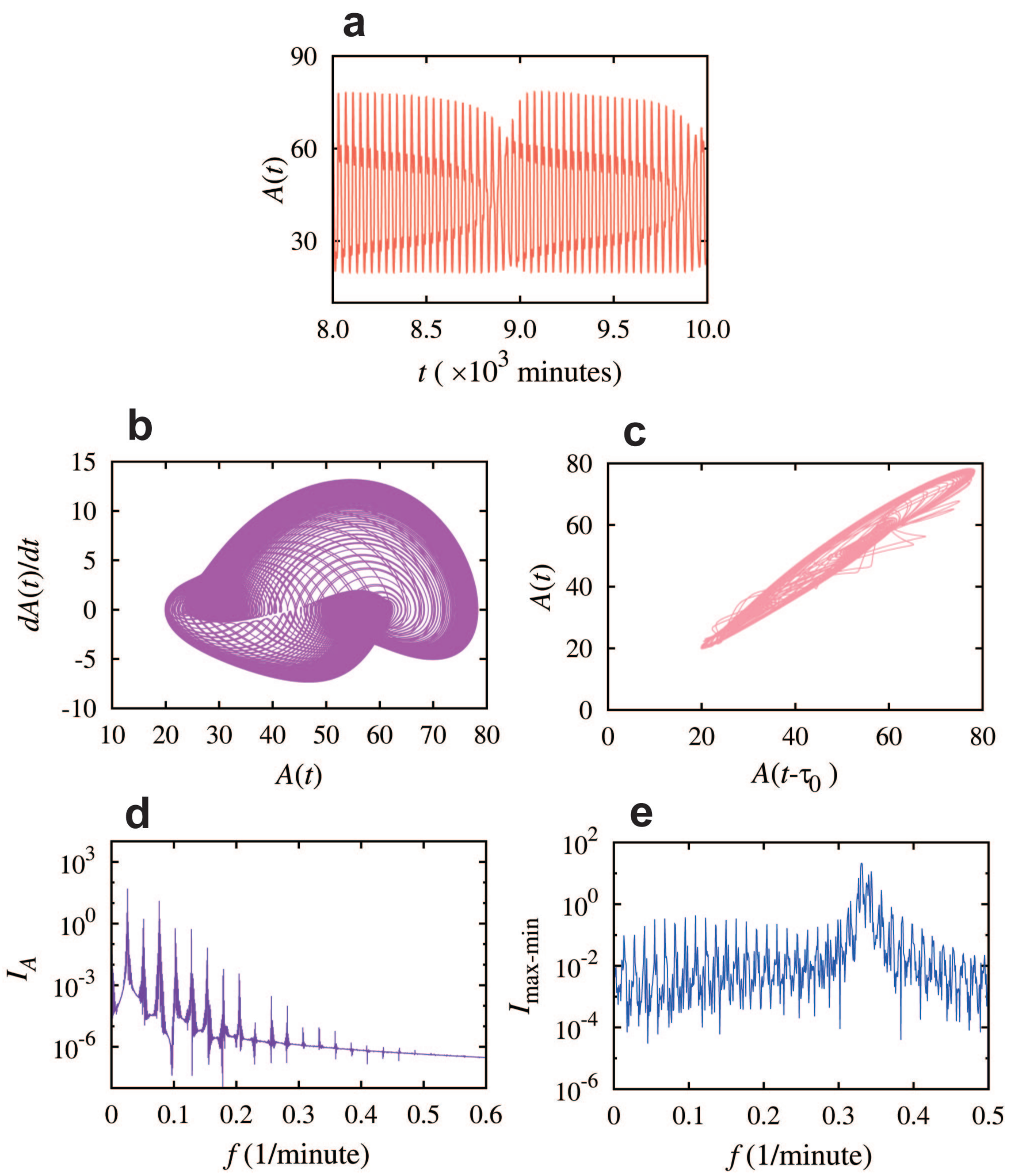

Figure SI2.3: **Weak chaotic dynamics of the one-gene element with two self-inhibitions.** Panels (a)-(e) show the dynamics of protein level $A(t)$ for $\tau_1 = 18$ minutes and τ_2 =3.113 minutes (green arrow in Fig. SI2.2(b)). Parameters are given by Table SI2.1. Panels (a), (b), and (c) show time trajectory, phase-space maps $A(t)$ - $dA(t)/dt$, and $A(t-\tau_0)$ - $A(t)$ (τ_0=39 minutes) respectively. Panels (d) and (e) are the full spectrum and the maximum-minimum spectrum respectively. The results suggest that the circuit dynamics is weak chaotic.

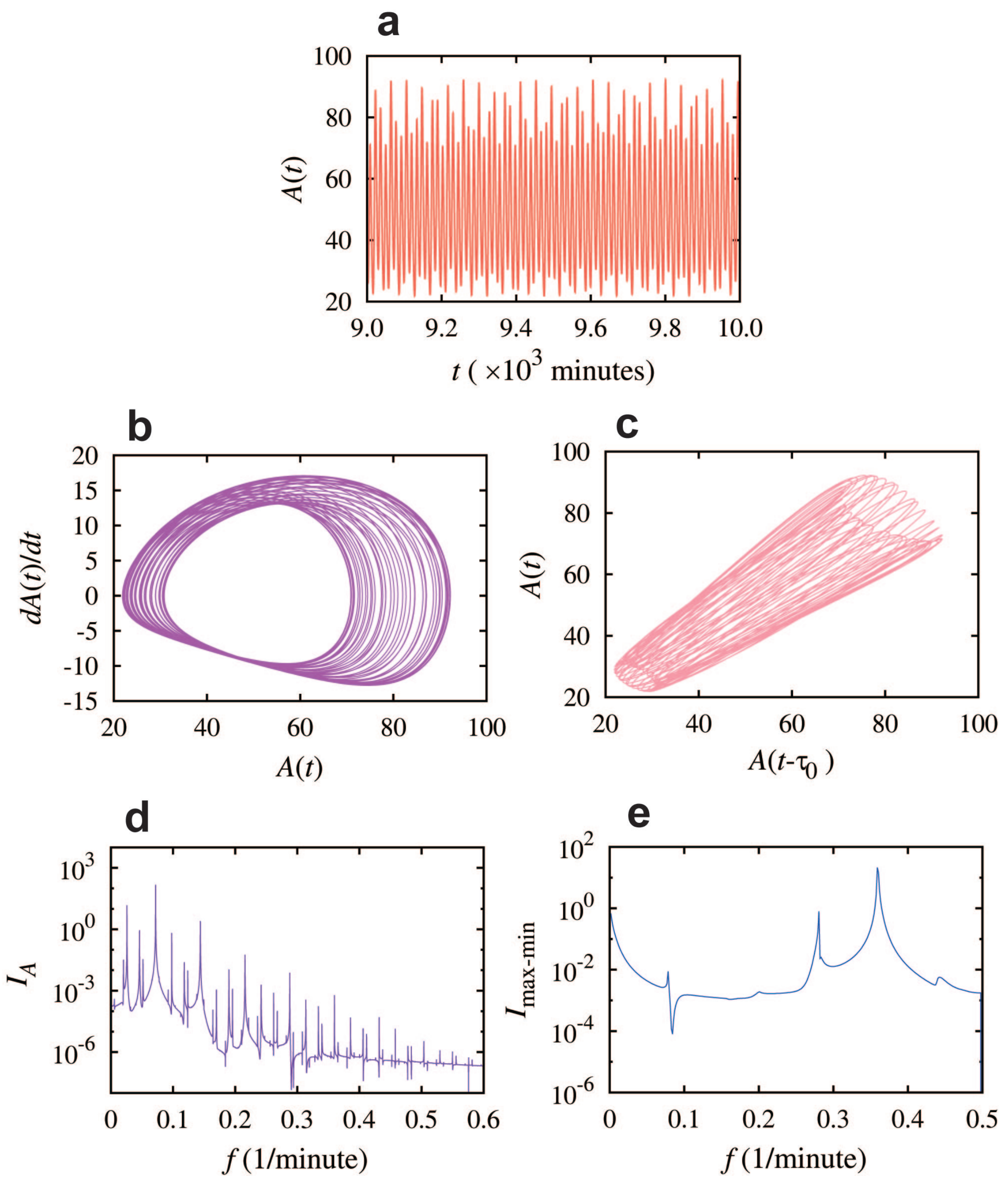

Figure SI2.4: **Quasi-periodic dynamics of the one-gene element with two self-inhibitions.** Panels (a)-(e) show the dynamics of protein level $A(t)$ for $\tau_1 = 18$ minutes and τ_2 =4.4 minutes (green arrow in Fig. SI2.2(c)). Parameters are given by Table SI2.1. Panels (a), (b), and (c) show time trajectory, phase-space maps $A(t)$ - $dA(t)/dt$ and $A(t-\tau_0)$ - $A(t)$ (τ_0=14 minutes) respectively. Panels (d) and (e) are the full spectrum and the maximum-minimum spectrum respectively. The results suggest that the circuit dynamics is quasi-periodic.

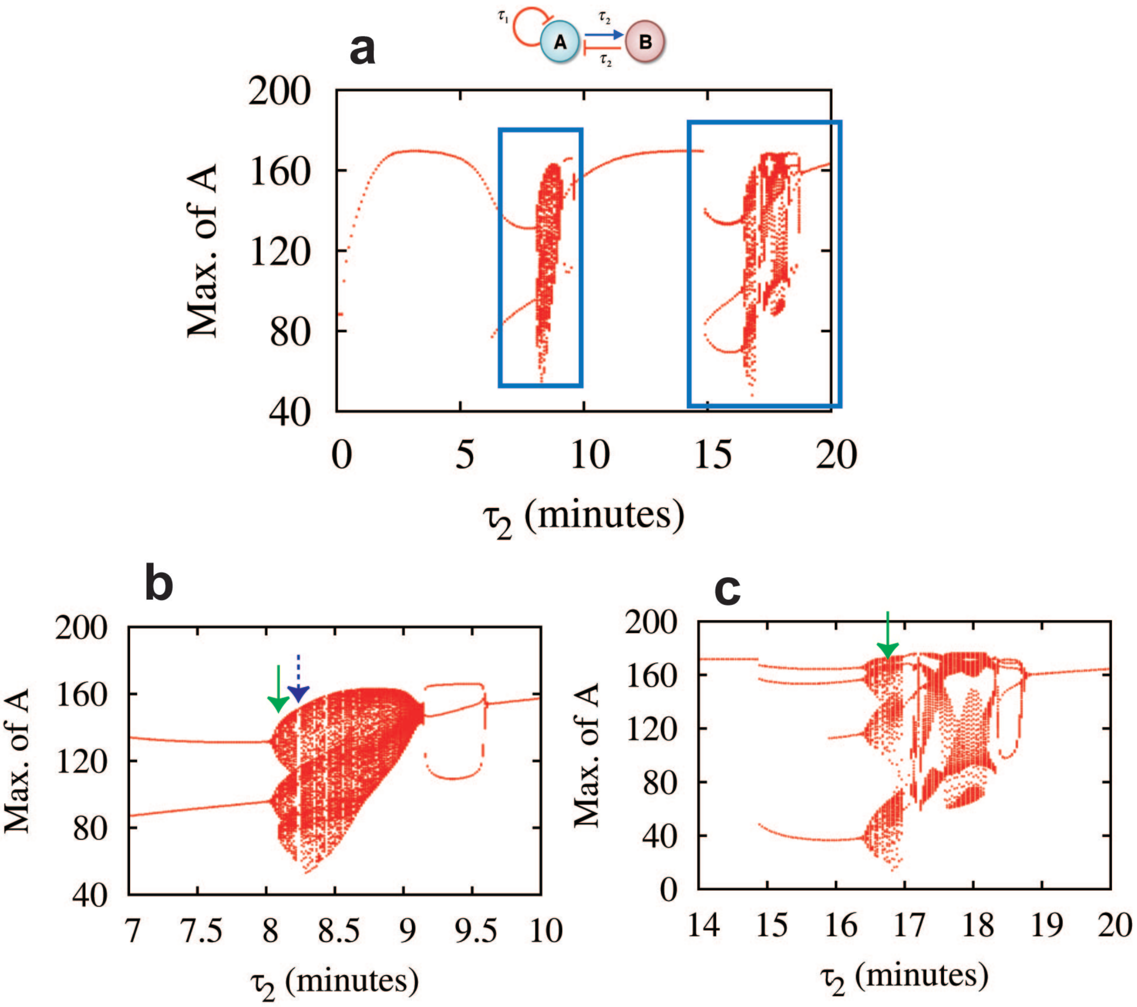

Figure SI2.5: **Bifurcation diagrams of the maximum levels of A for the two-gene flip-flop element with one-sided self-inhibition.** Panel (a) shows the bifurcation of the maximum levels of protein A (circuit diagram above panel (a)) with respect to the values of time delay τ_2 when the time delay τ_1 is set to be 5.3 minutes. In the bifurcation, non periodic dynamics are shown around $\tau_2 = 8.0$ minutes (left blue square in panel (a)) and around $\tau_2 = 17$ minutes (right blue square in panel (a)). The detailed bifurcations for these regions are shown in panels (b) and (c). The dynamics is non-periodic for 8.0 minutes $< \tau_2 <$ 9.18 minutes (panel (b)) and 16.5 minutes $< \tau_2 <$ 18.2 minutes (panel (c)). The circuit exhibits weak chaotic dynamics for $\tau_2 = 8.2$ minutes (blue arrow in panel (b)) as in Fig. 4 in the main article and Fig. SI2.1 and for $\tau_2 = 16.8$ minutes (green arrow in panel (c)) as in Fig. SI2.7. The circuit exhibits quasi-periodic for $\tau_2 = 8.05$ minutes (green arrow in panel (b)) as in Fig. SI2.6.

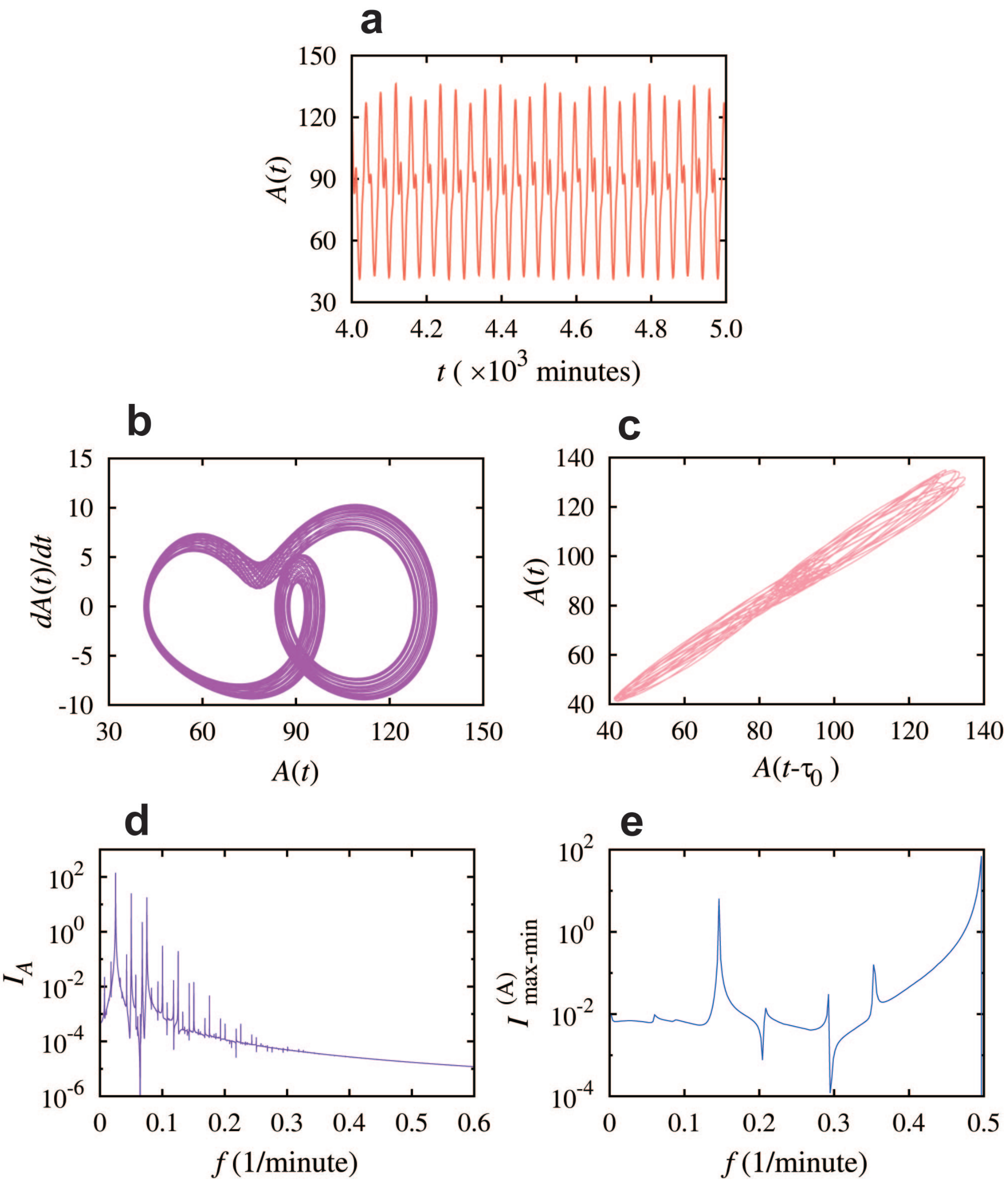

Figure SI2.6: **Quasi-periodic dynamics for the two-gene flip-flop element with one-sided self-inhibition.** Panels (a)-(e) show the dynamics of protein level for $\tau_1 = 5.3$ minutes and τ_2 =8.05 minutes (green arrow in Fig. SI2.5(b)). Parameters are given by Table SI2.2. Panels (a), (b), and (c) show time trajectory, phase-space maps $A(t)$ - $dA(t)/dt$ and $A(t-\tau_0)$ - $A(t)$ (τ_0=40 minutes) respectively. Panels (d) and (e) are the full spectrum and the maximum-minimum spectrum, respectively. The results suggest that the circuit dynamics is quasi-periodic. .

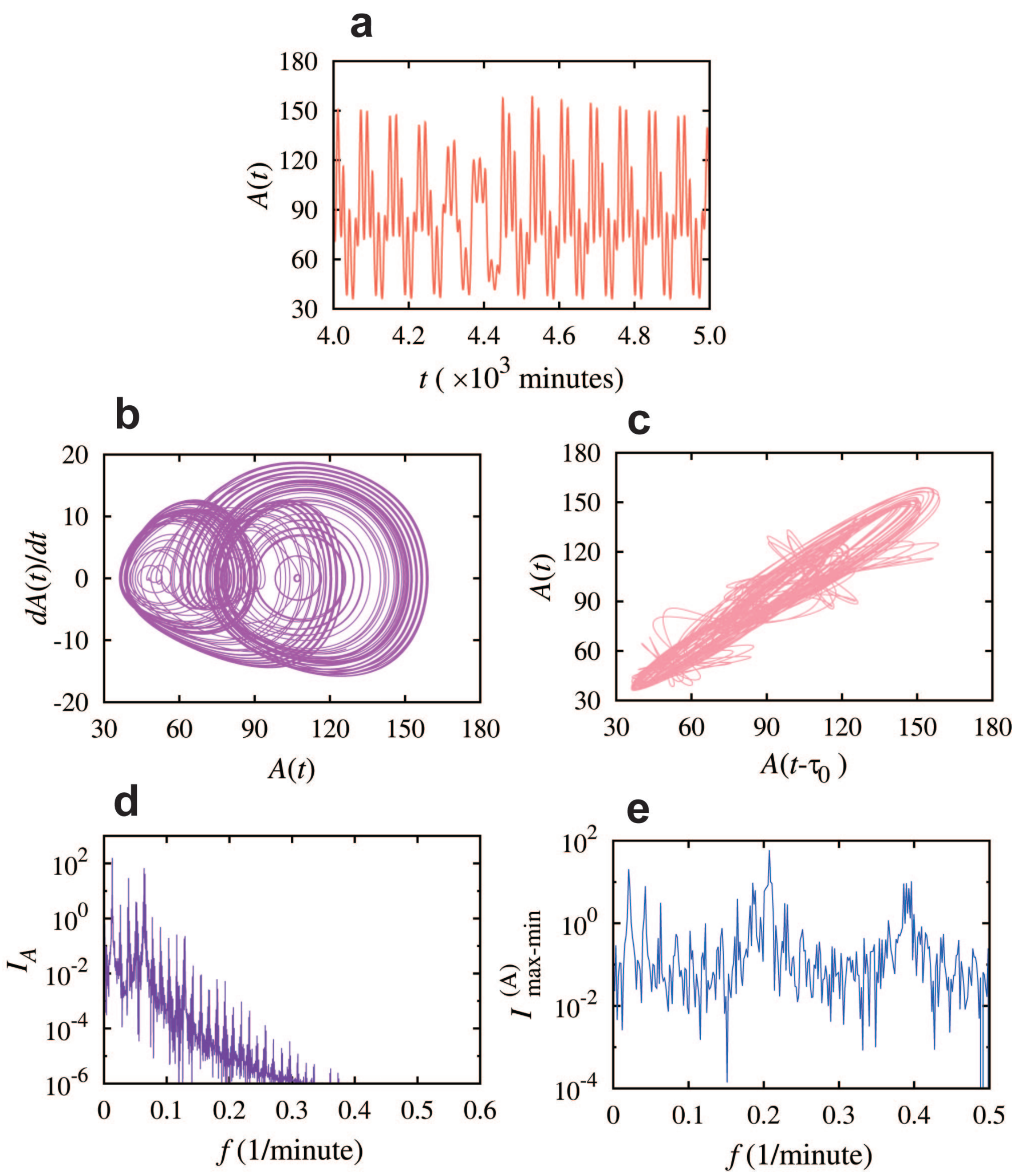

Figure SI2.7: **Weak chaotic dynamics for the two-gene flip-flop element with one-sided self inhibition.** Panels (a)-(e) show the dynamics of protein level for τ_1 = 5.3 minutes and τ_2 =16.8 minutes (green arrow in Fig. SI2.2(c)). Parameters are given by Table SI2.2. Panels (a), (b), and (c) show time trajectory, phase-space maps $A(t)$ - $dA(t)/dt$ and $A(t-\tau_0)$ - $A(t)$ (τ_0=77 minutes) respectively. Panels (d) and (e) are the full spectrum and the maximum-minimum spectrum respectively. The results suggest that the circuit dynamics is weak chaotic.

the circuit also has oscillatory dynamics but with a period of about 15 minutes instead (Fig. SI2.8(b)). The change in the period is caused by the time delay of the second feedback loop. In addition, we also observed oscillations with a period of about 40 minutes when $\tau_2 = 5$ minutes (Fig. SI2.8(c)) and $\tau_2 = 10$ minutes (Fig. SI2.8(d)). Interestingly, both the oscillations of the period of 15 minutes and 40 minutes are observed in the QP and WC modes, as shown in Fig. SI2.1(a), Fig. SI2.3(a), and Fig. SI2.4(a). The results suggest that, in the element, the first negative feedback loop is responsible for generating the oscillations with a period of about 40 minutes.

In order to check whether the oscillation with the period $T \simeq 15$ minutes in Fig. SI2.8(b) is related to the second negative feedback loop or not, we evaluate the dynamics for one-gene element with one delayed self-inhibition where the rank of Hill function $n = 2$ in Fig. SI2.9 (i.e., the second negative feedback loop). The circuit in Fig. SI2.9 is mostly same as the circuit of one-gene element with two delayed self-inhibitions except that the first negative feedback loop is removed. The bifurcation diagram of the fixed point and the maximum and minimum levels of protein A with respect to the time delay τ (Fig. SI2.9(a)) shows the Hopf bifurcation from the steady-state dynamics to the oscillatory dynamics occurs at $\tau_{\rm th} = 10.5$ minutes. The value of $\tau_{\rm th}$ is higher than the values of τ_2 in Fig. SI2.8(a)-(d). The period of the oscillation for $\tau = 18$ minutes is around 40 minutes (Fig. SI2.9(b)). The result suggests that the oscillation with period $T \simeq 15$ minutes is not caused by the second feedback loop itself.

Next, we evaluate the change of the circuit dynamics caused by the second feedback loop for the other values of τ_1. Figures SI2.10(a)-(d) show the bifurcation diagrams of the fixed point and the maximum and minimum levels of protein A with respect to τ_2 for different τ_1. When there is no time delay in the first negative feedback loop, i.e., $\tau_1 = 0.0$ minutes (Fig. SI2.10(a)), the circuit exhibits dynamics of steady state, no matter if we introduce time delay in the second negative feedback loop or not. The results demonstrate the essential role of the delay in the first negative feedback loop in generating non-steady-state dynamics.

When $\tau_1 = 3.0$ minutes (Fig. SI2.10(b)) and $\tau_1 = 4.0$ minutes (Fig. SI2.10(c)), the circuit exhibits oscillatory dynamics for certain values of τ_2. There are several bifurcation points for the switches between steady-state dynamics and oscillatory dynamics. Among them, the smallest bifurcation point is at about $\tau_2 = 3.0$ minutes. When $\tau_1 = 15$ minutes (Fig. SI2.10(d)), the circuit always exhibits oscillatory dynamics, no matter what τ_2 is selected. Again, at about $\tau_2 = 3.0$ minutes, the dynamics is different in that it contains two mixed oscillations. The values of τ_2 coincides with the bifurcation point of the previous cases, and it also coincides with the conditions for weak chaotic dynamics. Therefore, the results suggest that the second feedback loop allows flipping between two dynamic modes (the steady-state dynamics and the oscillatory dynamics), since the value of time delay is close to the bifurcation point.

In summary, we conclude that, this minimalist chaotic element requires a first negative feedback loop to generate stable oscillations and a second negative feedback loop to allow switches between two dynamic modes. We further hypothesize that this might be also true for many other chaotic motifs, which worth further investigation.

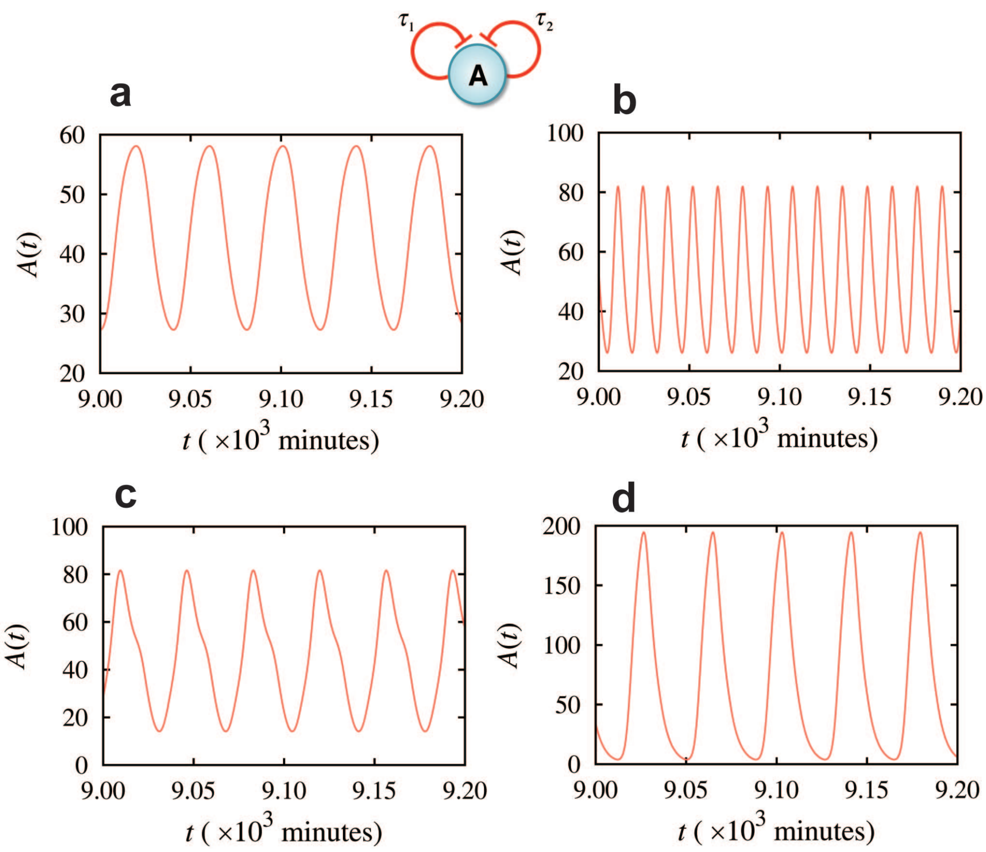

Figure SI2.8: **Oscillatory dynamics for the one-gene element with two delayed self-inhibitions.** When $\tau_1 = 18$ minutes, the circuit exhibits oscillatory dynamics for a wide range of τ_2, as also shown in the bifurcation diagram in Fig. SI2.2. However, the period of the oscillations depends on the value of τ_2. When there is no delay in the second negative feedback loop ($\tau_2 = 0.0$ minutes, panel (a)), the period of the oscillation is around 40 minutes; when $\tau_2 = 4.0$ minutes (panel (b)), the period is around 15 minutes instead. When $\tau_2 = 5.0$ minutes (panel (c)) and $\tau_2 = 10$ minutes (panel (d)), the period of the oscillation is around 40 minutes, again.

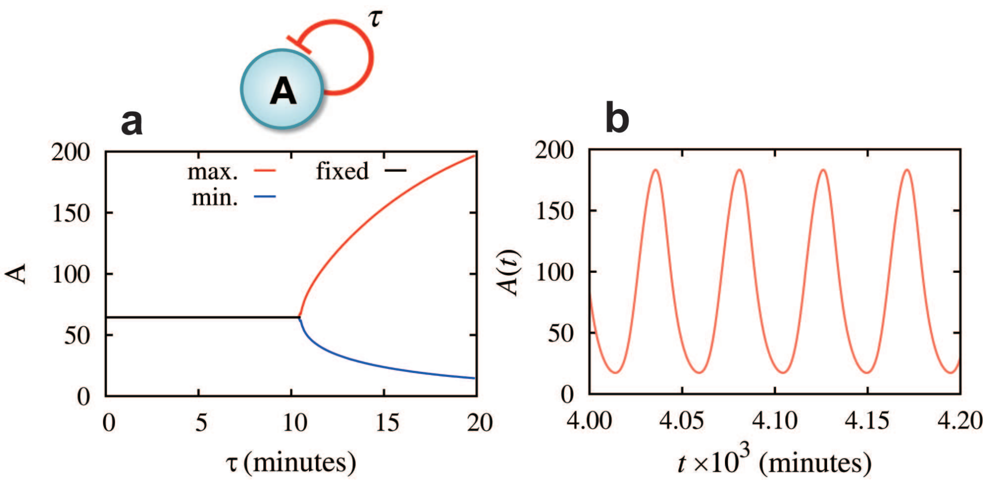

Figure SI2.9: **Oscillatory dynamics for the one-gene element with one delayed self-inhibition where the rank of Hill function $n = 2$.** Here, the circuit is mostly same as the circuit shown in Fig. SI2.8 except that the first negative feedback loop is removed. Panel (a) shows the bifurcation diagram of A with respect to the value of the time delay τ. If the circuit exhibits steady-state dynamics, the diagram shows the values of A in the steady states in black; if the circuit exhibits oscillatory dynamics, the diagram shows both the maximum (red) and minimum(blue) for each oscillation, respectively. The Hopf bifurcation point from steady-state dynamics to oscillatory dynamics occurs at time delay $\tau_{\text{th}} = 10.5$ minutes. Panel (b) shows the oscillatory dynamics for the time delay $\tau = 18$ minutes. The period of oscillation is around 40 minutes.

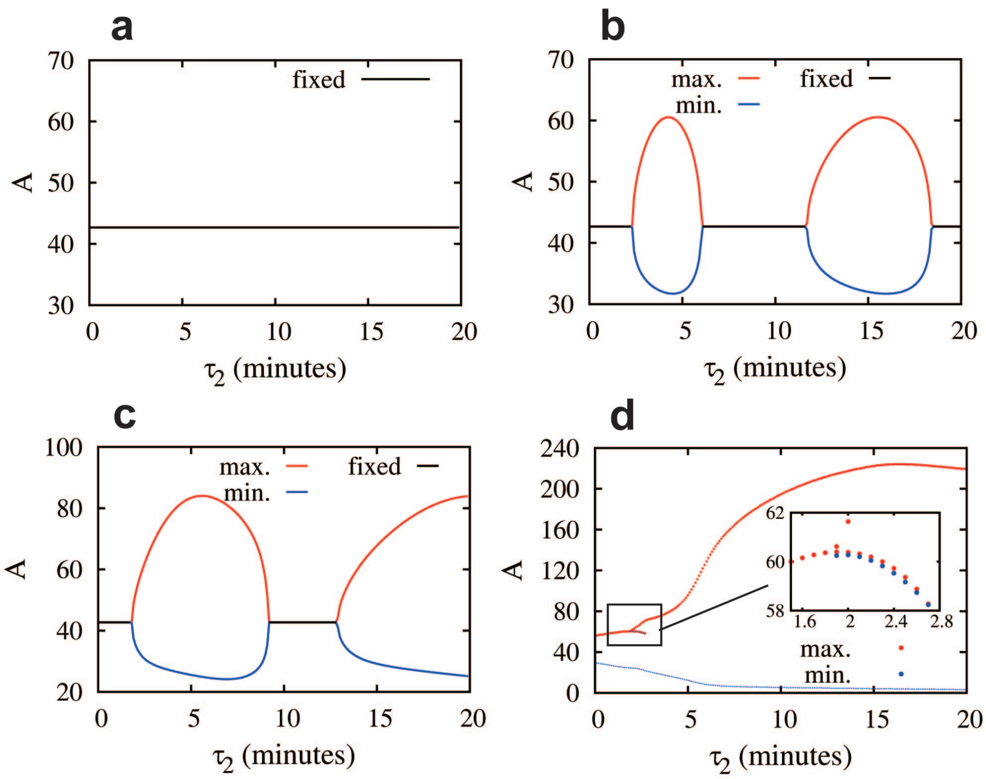

Figure SI2.10: **Bifurcations for the one-gene element with two delayed self-inhibitions.** Panels (a)-(d) show bifurcation diagrams of A levels with respect to τ_2 for different values of τ_1. If the circuit exhibits steady-state dynamics, the diagram shows the values of A in the steady states in black; if the circuit exhibits oscillatory dynamics, the diagram shows both the maximum (red) and minimum(blue) for each oscillation, respectively. When there is no time delay in the first negative feedback loop ($\tau_1 = 0.0$ minutes, panel (a)), the circuit only exhibits steady-state dynamics. When $\tau_1 = 3.0$ minutes (panel (b)) and $\tau_1 = 4.0$ minutes (panel (c)), the circuit exhibits Hopf bifurcation from steady-state dynamics to oscillatory dynamics when τ_2 increases from 0 minute to about 3 minutes. The Hopf bifurcation point (the left-most one) is $\tau_2 = 2.3$ minutes for (b) and $\tau_2 = 1.8$ minutes for (c). When $\tau_1 = 15$ minutes (panel (d)), the circuit always exhibits oscillatory dynamics. Interestingly, around $\tau_2 \simeq 2$ minutes, the circuit dynamics switches to a mode of two mixed oscillations (inset for a zoom-in diagram).

SI 3 Elements with two negative feedbacks and one positive feedback

SI3.1 Models and parameters (Fig. 5)

In this section, we show the details for modeling elements with two negative feedbacks and one positive feedback in Fig. 5. Table SI3.1 shows model parameters of the the single gene element with two self-inhibitions (with time delays τ_1, τ_2) and one self-activation (with time delay τ_3).

Table SI3.1: **Model parameters for the single gene element with two self-inhibitions and one self-activation (Eq. (4)) in the main article.**

Parameter	Value
g_A	50 (nM/minute)
k_A	0.20 (minute^{-1})
$A_{0,1AA}$	38 (nM)
$A_{0,2AA}$	38 (nM)
$A_{0,3AA}$	28 (nM)
n_{1AA}	4
n_{2AA}	4
n_{3AA}	4

The deterministic rate equation for the the two-gene circuit with two self-inhibitory and mutually activating genes (the inset of Fig. 5d in the main article) is described by

$$\frac{dA(t)}{dt} = [g_A + g_{AB}B(t-\tau_{12})]H^{-}_{AA}[A(t-\tau_1)] - k_A A(t), \quad \text{(SI3.1)}$$

$$\frac{dB(t)}{dt} = [g_B + g_{BA}A(t-\tau_{21})]H^{-}_{BB}[B(t-\tau_2)] - k_B B(t), \quad \text{(SI3.2)}$$

where the mutual activations are modeled by linear functions. Model parameters in Eqs. (SI3.1) and (SI3.2) are given in Table SI3.2.

SI3.2 Strong chaotic dynamics

We here show additional results of the strong chaotic dynamics described in Fig. 5. The left panels of Fig. SI3.1 are results for the single-gene element with two self-inhibitions and one self-activation, where the time delays are set to $\tau_1 = 18.0$ minutes, $\tau_2 = 8.0$ minutes, and $\tau_3 = 12.5$ minutes. The right panels of Fig. SI3.1 are results for the the two-gene circuit with two self-inhibitory and mutually activating genes, where the time delays $\tau_1 = 6.0$ minutes, $\tau_2 = 5.0$ minutes, $\tau_{12} = 7.5$ minutes and $\tau_{21} = 16.0$ minutes. (a) and (d) show time trajectories. (b) and (e) show phase-space maps $A(t-\tau_0)$ - $A(t)$. The value of τ_0 is given by $1/f_0$ where f_0 is the frequency of the maximum point of the full

Table SI3.2: **Model parameters for the two-gene circuit with two self-inhibitory and mutually activating genes described by Eqs. (SI3.1) and (SI3.2).**

Parameter	Value
g_A	50 (nM/minute)
g_B	50 (nM/minute)
k_A	0.20 (minute^{-1})
k_B	0.24 (minute^{-1})
$A_{0,AA}$	100 (nM)
$B_{0,BB}$	100 (nM)
n_{AA}	4
n_{BB}	4
g_{AB}	3.0 (1/minute)
g_{BA}	3.0 (1/minute)

spectrum (Figs. 5(c) and (f) in the main article). Here we set τ_0 to be 15.18 minutes for (b) and 28.69 minutes for (e). (c) and (f) show the maximum-minimum spectra.

From the results of both Fig. 5 in the main article and Fig. SI3.1, we conclude that strong chaotic dynamics can emerge for the circuits shown in the insets of Figs. 5(a) and (d).

SI3.3 Some other possible dynamical behaviors

Other than strong chaotic dynamics, these elements can also exhibit some other non-periodic dynamics. For example, we show that the one-gene element with two self-inhibitions and one self-activation can have quasi-periodic motion, as shown in the left panels in Fig. 2 in the main article and the panels in Fig. SI3.2. Here, the time delays τ_1 = 18.0 minutes, τ_2 = 8.0 minutes, and τ_3 = 18.5 minutes. The rest parameters are listed in Table SI3.1.

Similarly, both quasi-periodic and weak chaotic dynamics are observed for the two-gene circuit with two self-inhibitory and mutually activating genes, as shown in Fig. SI3.3 and Fig. SI3.4, respectively. The time delays are τ_1 = 6.0 minutes, τ_2 = 5.0 minutes, and τ_{12} = 7.5 minutes. We set τ_{21} = 13.56 minutes for Fig. SI3.3 and τ_{21} = 13.8 minutes for Fig. SI3.4.

As mentioned in the results in the main article, if the mutual activations are modeled by excitatory Hill functions, the circuit can also exhibit weak chaotic dynamics. The deterministic rate equation is given by

$$\frac{dA(t)}{dt} = g_A H^{+}_{AB}[B(t-\tau_{12})] H^{-}_{AA}[A(t-\tau_1)] - k_A A(t), \tag{SI3.3}$$

$$\frac{dB(t)}{dt} = g_B H^{+}_{BA}[A(t-\tau_{21})] H^{-}_{BB}[B(t-\tau_2)] - k_A B(t). \tag{SI3.4}$$

An example of weak chaotic dynamics of this circuit is shown in Fig. SI3.5. The corresponding parameters are given by Table SI3.3.

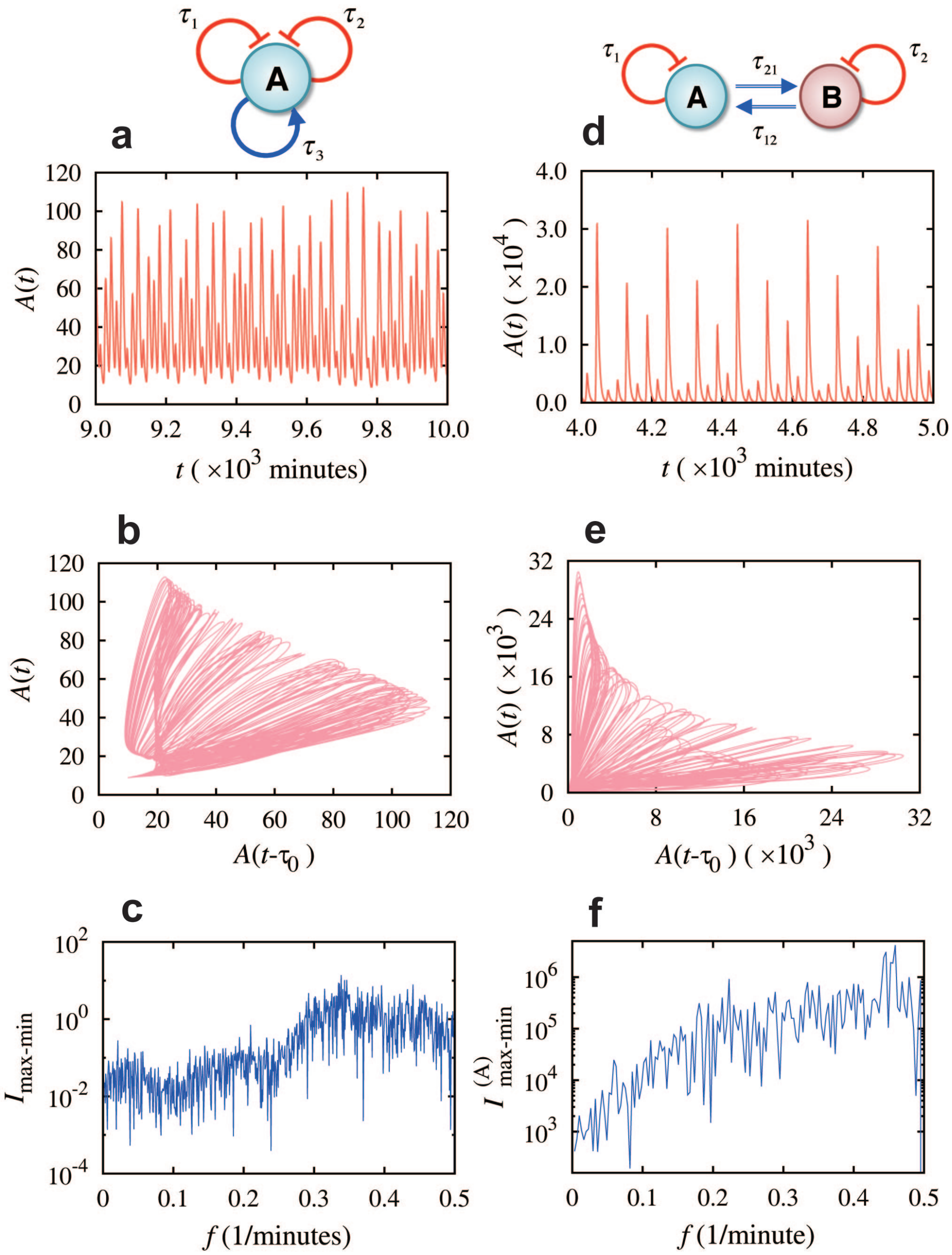

Figure SI3.1: **Strong chaotic dynamics in the circuit motifs with one positive feedback and two negative feedback loops.** Left panels show the results for the one-gene (A) element with two self-inhibitions and one self-activation (circuit diagrams above panel (a)); right panels for the two-gene (A and B) circuit with two self-inhibitory and mutually activating genes (circuit diagrams above panel (d)). The time delays are set to be the same values as those in Fig. 5 in the main article, i.e. $\tau_1 = 18.0$ minutes, $\tau_2 = 8.0$ minutes, and $\tau_3 = 12.5$ minutes in the left panels and $\tau_1 = 6.0$ minutes, $\tau_2 = 5.0$ minutes, $\tau_{12} = 7.5$ minutes, and $\tau_{21} = 16$ minutes in the right panels. (a) and (d) show time trajectories. (b) and (e) show the phase-space maps $A(t - \tau_0)$ - $A(t)$, where τ_0 is set to be 15.18 minutes in (b) and 28.69 minutes in (e). (c) and (f) show the maximum-minimum spectra of $A(t)$.

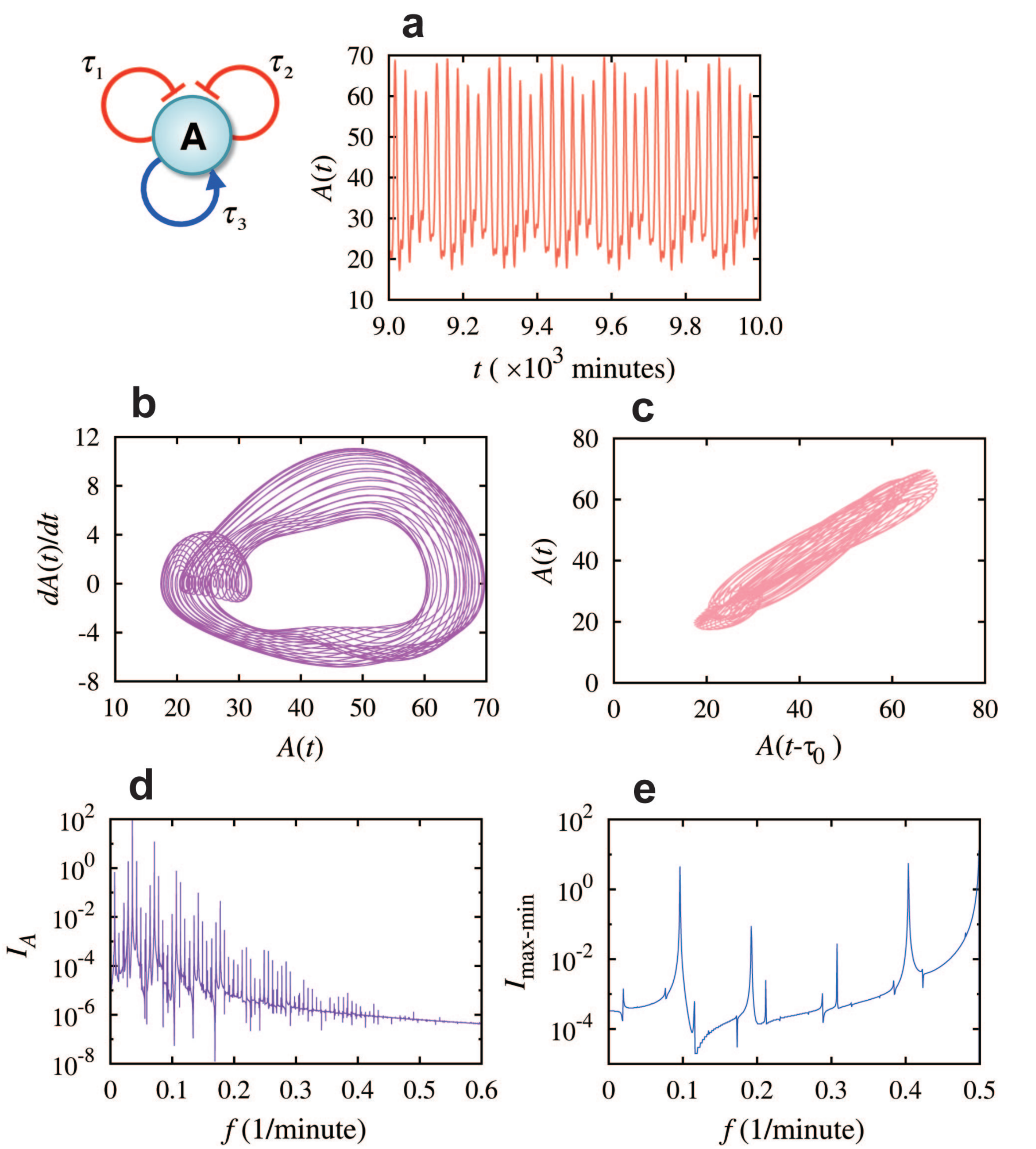

Figure SI3.2: **Quasi-periodic dynamics for the one-gene element with two self-inhibitions and one self-activation.** Here we chose the time delays $\tau_1 = 18.0$ minutes, $\tau_2 = 8.0$ minutes, and $\tau_3 = 18.5$ minutes. The rest parameters are listed in Table SI3.1. Panels (a), (b), and (c) show the time trajectory, the phase-space maps $A(t)$ - $dA(t)/dt$ and $A(t-\tau_0)$ - $A(t)$, where τ_0=28.17 minutes. Panels (d) and (e) are the full spectrum and the maximum-minimum spectrum. The results suggest that the circuit dynamics is quasi-periodic. .

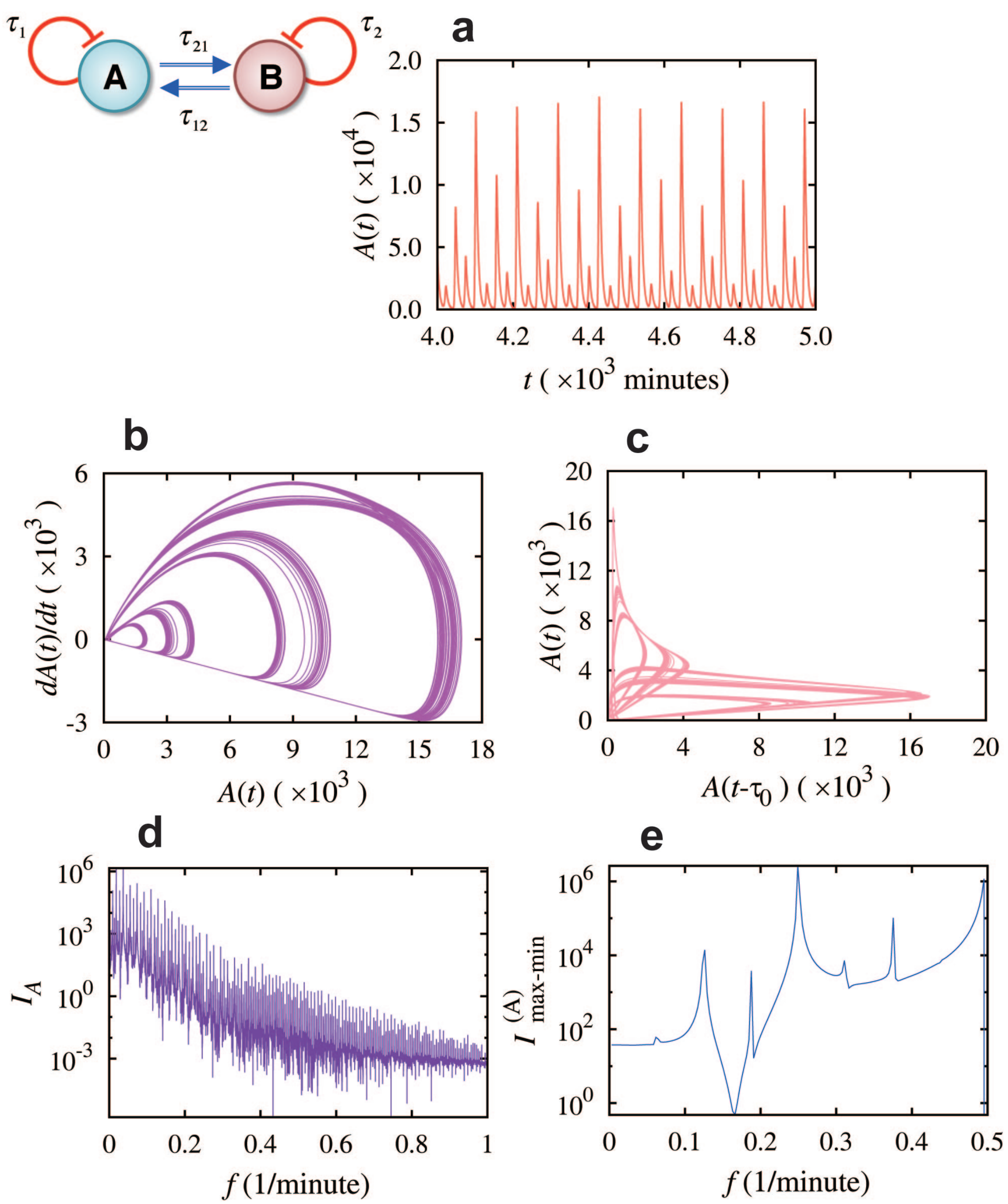

Figure SI3.3: **Quasi-periodic dynamics for the two-gene circuit with two self-inhibitory and mutually activating genes.** Here we chose the time delays τ_1 = 6.0 minutes, τ_2 = 5.0 minutes, τ_{12} = 7.5 minutes, and τ_{21} = 13.56 minutes. The rest parameters are listed in Table SI3.2. Panels (a), (b), and (c) show the time trajectory, the phase-space maps $A(t)$ - $dA(t)/dt$ and $A(t-\tau_0)$ - $A(t)$, where τ_0=23.13 minutes. (d) is the full spectrum and (e) is the maximum-minimum spectrum. The results suggest that the circuit dynamics is quasi-periodic.

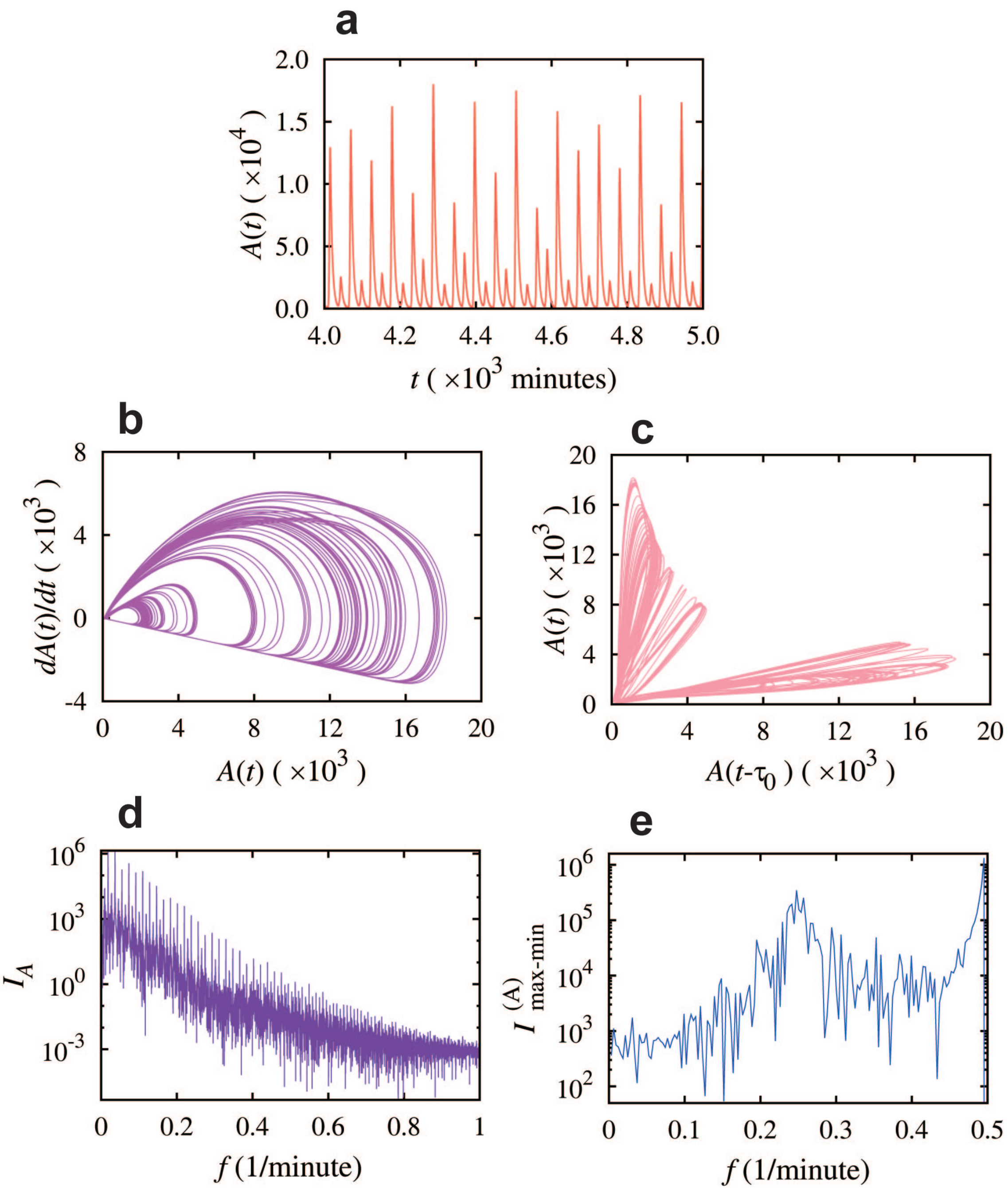

Figure SI3.4: **Weak-chaotic dynamics for the two-gene circuit with two self-inhibitory and mutually activating genes.** Here we chose the time delays $\tau_1 = 6.0$ minutes, $\tau_2 = 5.0$ minutes, $\tau_{12} = 7.5$ minutes, and $\tau_{21} = 13.8$ minutes. The rest parameters are listed in Table SI3.2. Panels (a), (b), and (c) show the time trajectory, the phase-space maps $A(t)$ - $dA(t)/dt$ and $A(t - \tau_0)$ - $A(t)$, where τ_0=27.24 minutes. (d) is the full spectrum and (e) is the maximum-minimum spectrum. The results suggest that the circuit dynamics is weak chaotic.

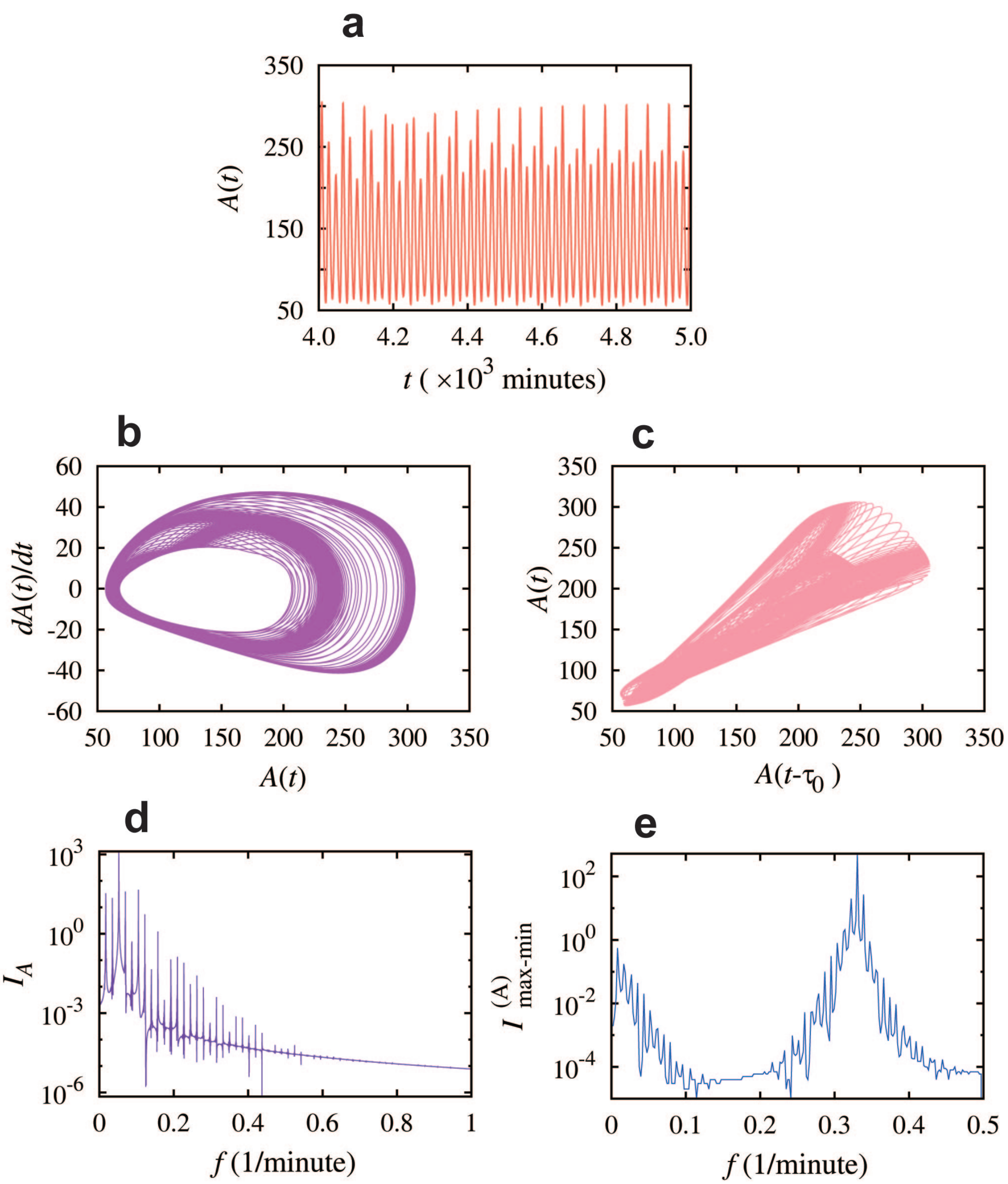

Figure SI3.5: **Weak chaotic dynamics for the two-gene circuit with two self-inhibitory and mutually activating genes where the activations are modeled by excitatory Hill functions.** Here we chose the time delays $\tau_1 = 6.0$ minutes, $\tau_2 = 5.0$ minutes, $\tau_{12} = 7.5$ minutes, and $\tau_{21} = 12.15$ minutes. The rest parameters are listed in Table SI3.3. Panels (a), (b), and (c) show the time trajectory, the phase-space maps $A(t)$ - $dA(t)/dt$ and $A(t-\tau_0)$ - $A(t)$, where τ_0=19.07 minutes. (d) is the full spectrum and (e) is the maximum-minimum spectrum. The results suggest that the circuit dynamics is weak chaotic.

Table SI3.3: **Model parameters for the two-gene circuit with two self-inhibitory and mutual activating genes described by Eqs. (SI3.3) and (SI3.4).**

Parameter	Value
g_A	150 (nM/minute)
g_B	150 (nM/minute)
k_A	0.20 (minute^{-1})
k_B	0.31 (minute^{-1})
$A_{0,AA}$	100 (nM)
$B_{0,BB}$	100 (nM)
$A_{0,BA}$	100 (nM)
$B_{0,AB}$	100 (nM)
n_{AA}	4
n_{BB}	4
n_{BA}	1
n_{AB}	1

SI 4 P-SC intermittent dynamics in the one-gene element with both self-activation and self-inhibition

SI4.1 Models and parameters in Figs. 6 and 7

The parameters for one-gene element with both self-activation and self-inhibition (Eq. (5) in the main article) are given by Table SI4.1. For the parameter set, the circuit exhibits the intermittency between periodic and strong chaotic mode.

Table SI4.1: **Model parameters for the one-gene element with both self-inhibition and self-activation (Eq. (5)) in the main article.**

Parameter	Value
g_A	50 (nM/minute)
k_A	0.20 (minute^{-1})
$A_{0,1AA}$	38 (nM)
$A_{0,2AA}$	38 (nM)
n_{1AA}	6
n_{2AA}	1

SI4.2 Additional results of the circuit

We here show additional results of the intermittent dynamics in Figs. 6 and 7. The bifurcation diagram of maximum levels of protein A is shown in Fig. 6(a) in the main article. Fig. SI4.1 shows the phase space map, $A(t-T)$ - $A(t)$, where T is the period of the periodic dynamics. The values of time delays τ_1 and τ_2 are same as those for the simulation shown in Fig. 6 in the main article. The time delay for the self-inhibitory regulation τ_1 is set to be 26 minutes. The time delay for the self-excitatory regulation τ_2 is set to be 26.3 minutes in (a), 26.29 minutes in (b), and 26.0 minutes in (c).

The green line in the panel (a) shows that $A(t)$ always equals to $A(t-T)$ (the period $T = 1.0131 \times 10^4$ minutes), indicating periodic oscillatory dynamics. The red line in the panel (c) indicates chaotic dynamics. The trajectory in the panel (b) contains both the periodic dynamics (green) and the chaotic dynamics (red), indicating the coexistence of both modes. Here T is set to be 1.0149×10^4 minutes in the panels (b) and (c).

In Fig. SI4.2, the time delay τ_2 is set to be 26.25 minutes in (a), and 26.29 minutes in (b). Fig. SI4.2 indicates that the closer the τ_2 to the bifurcation point around $\tau_2 =$ 26 minutes (Fig. 6(a) in the main article), the longer the time intervals (illustrated by the green arrows) of the periodic dynamics; meanwhile the duration (illustrated by the magenta arrows) of the SC dynamics remains the same, as mentioned in the results in the main article.

Additional results are shown in Fig. SI4.3 for $\tau_2 = 26.25$ minutes, where the dynamics belong to the intermittency between periodic and strong chaotic behaviors.

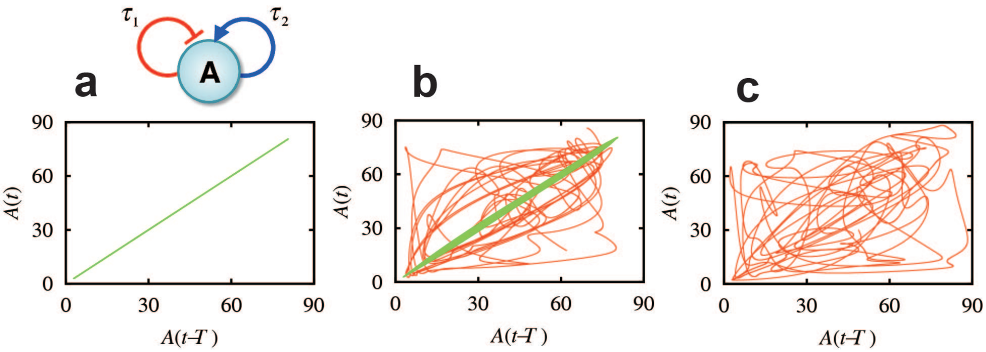

Figure SI4.1: **The phase diagram $A(t-T)$ - $A(t)$ for the one-gene element with both self-activation and self-inhibition.** The time delay of the self-inhibition τ_1 is set to be 26 minutes. The time delay of the self-activation τ_2 is set to be 26.3 minutes, 26.29 minutes, and 26.0 minutes for (a), (b), and (c) respectively. In (a), T is set to be the period of the oscillation 1.0131×10^4 minutes. In (b) and (c), T is set to be approximately the period of the oscillation in (b) 1.0149×10^4 minutes.

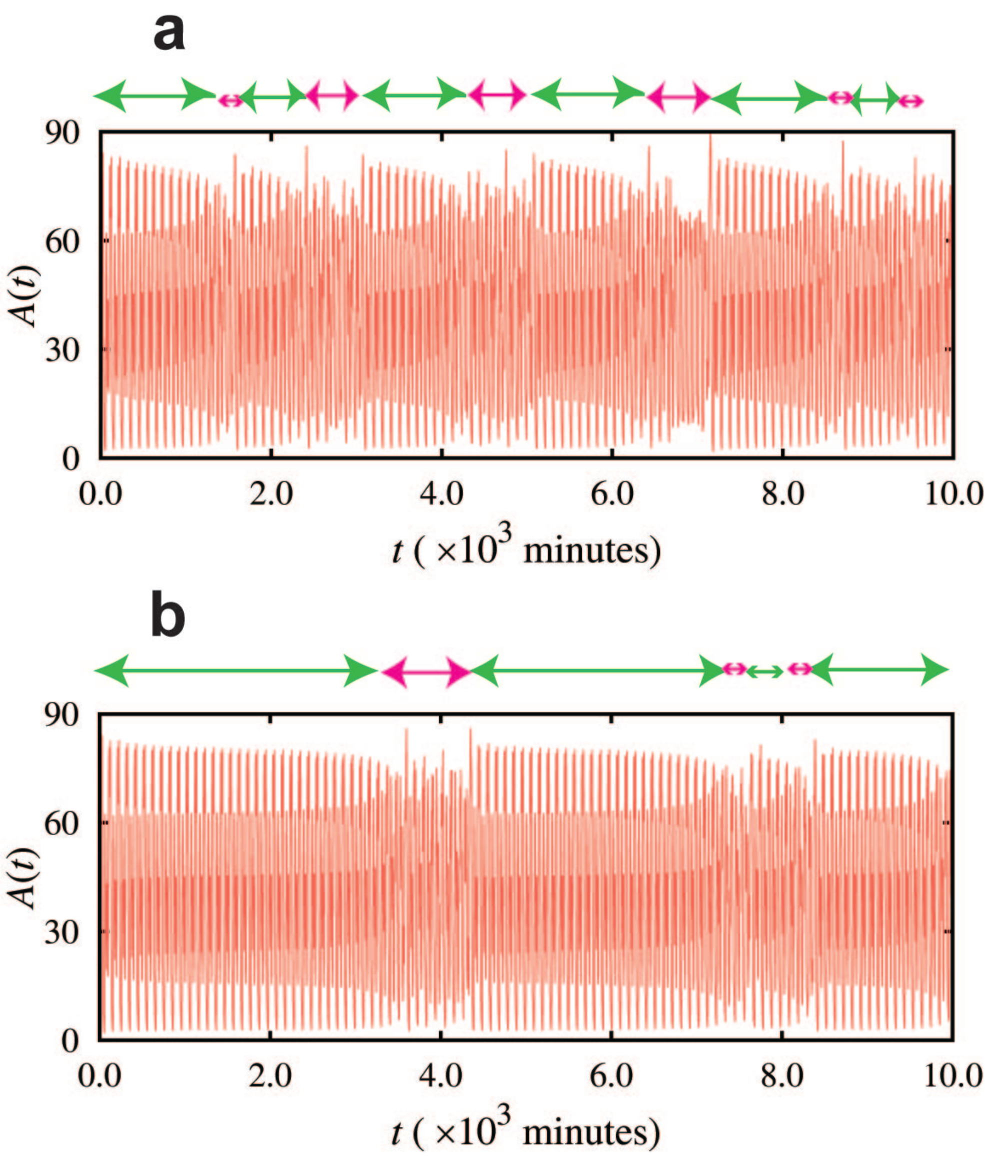

Figure SI4.2: **Time dynamics of the intermittency between periodic and strong chaotic modes for the circuit element with both self-activation and self-inhibition.** The time delay of the self-inhibition τ_1 is set to be 26 minutes. The time delay τ_2 is set to 26.25 minutes and 26.29 minutes in the panels (a) and (b) respectively, From the bifurcation diagram (Fig. 6(a) in the main article), the value of τ_2 in (b) is closer to the bifurcation point around $\tau_2 = 26$ minutes than that in (a). The time intervals (green arrows) of the periodic dynamics in (a) are shorter than those in (b), while the duration (magenta arrows) of the SC dynamics in (a) is same as that in (b).

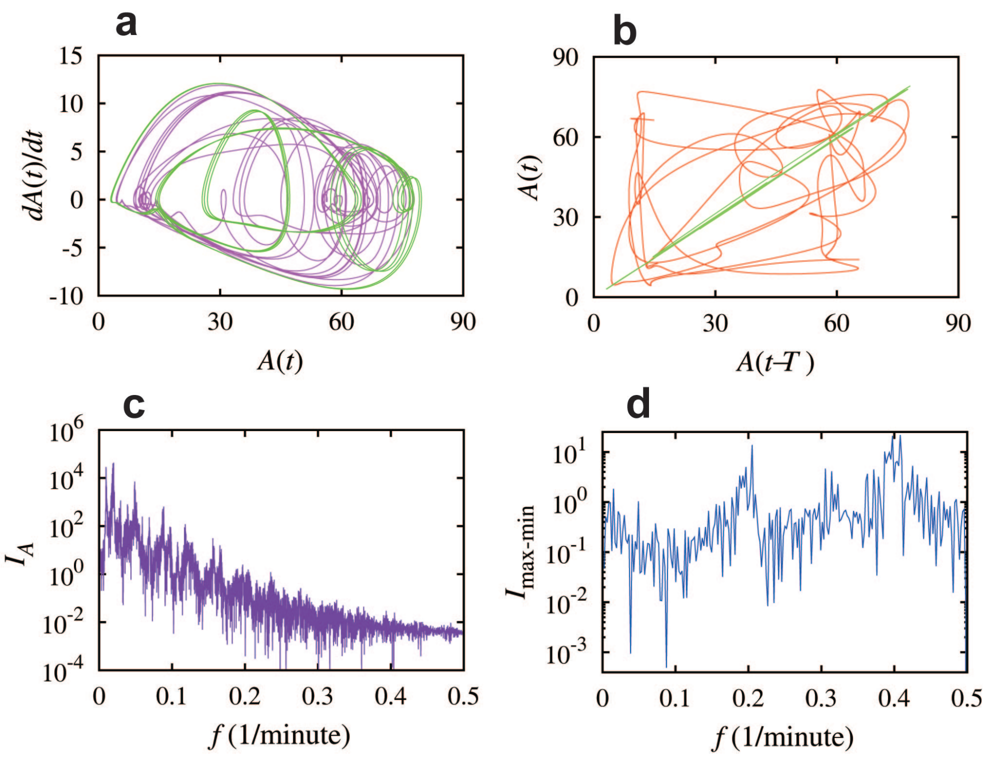

Figure SI4.3: **Additional dynamical behaviors corresponding to the panel Fig. SI4.2(a).** (a) and (b) show the phase-space maps $A(t)$ - $dA(t)/dt$ and $A(t-T)$ - $A(t)$ respectively. T is set to be 1.0193×10^4 minutes. The green lines in (a) and (b) show the periodic oscillatory dynamics, while the purple line in (a) and the red line in (b) show the chaotic dynamics. These two dynamics coexist in the maps in (a) and (b). (c) and (d) are the full and maximum-minimum spectra respectively. They indicate that the non-periodic part of the time trajectory in Fig. SI4.2(a) is strong chaos.

SI 5 WC-SC intermittent dynamics in the one-gene element with two self-inhibitions and one self-activation

As mentioned in the results in the main article, WC-SC intermittent dynamics are observed in the one-gene element with two self-inhibitions and one self-activation (upper circuit element in Fig. 1(c)). We used the same parameters as in Table. SI3.1 in SI3. The time delays of the self-inhibitions τ_1 and τ_2 are set to be 18 minutes and 8 minutes respectively.

Fig. SI5.1(a) is the bifurcation diagram of maximum levels of protein A with respect to τ_3. Intermittency between SC and WC modes emerges around $\tau_3 = 13$ minutes. When the time delay of the self-activation τ_3 is set to be 13.3 minutes, the circuit element exhibits weak chaotic dynamics, as shown in Fig. SI5.1(b), (c), Fig. SI5.2(a), and Fig. SI5.3(a), (b). When the time delay of the self-activation τ_3 is set to be 12.5 minutes, the circuit element exhibits strong chaotic dynamics, as shown in Fig. SI5.1(f), (g), Fig. SI5.2(c), and Fig. SI5.3(e), (f).

When the time delay of the self-activation τ_3 is set to be 13.2 minutes, the circuit element exhibits the intermittency between the weak and strong chaotic behaviors, as shown in Fig. SI5.1(d). Moreover, the phase-space maps, $A(t)$ - $dA(t)/dt$ (Fig. SI5.1 (e)) and $A(t - \tau_0)$ - $A(t)$ (Fig. SI5.2(b)) indicate the coexistence of weak chaotic dynamics (green in Figs. SI5.1(e) and SI5.2(b)) and strong chaotic dynamics (purple in Fig. SI5.1(e) and red in Fig. SI5.2(b)). The full spectrum for the intermittent dynamics (Fig. SI5.3(c)) has a mixture of both up and downward spikes, indicating again the mixture of the behaviors of both the weak chaotic dynamics and the strong chaotic dynamics.

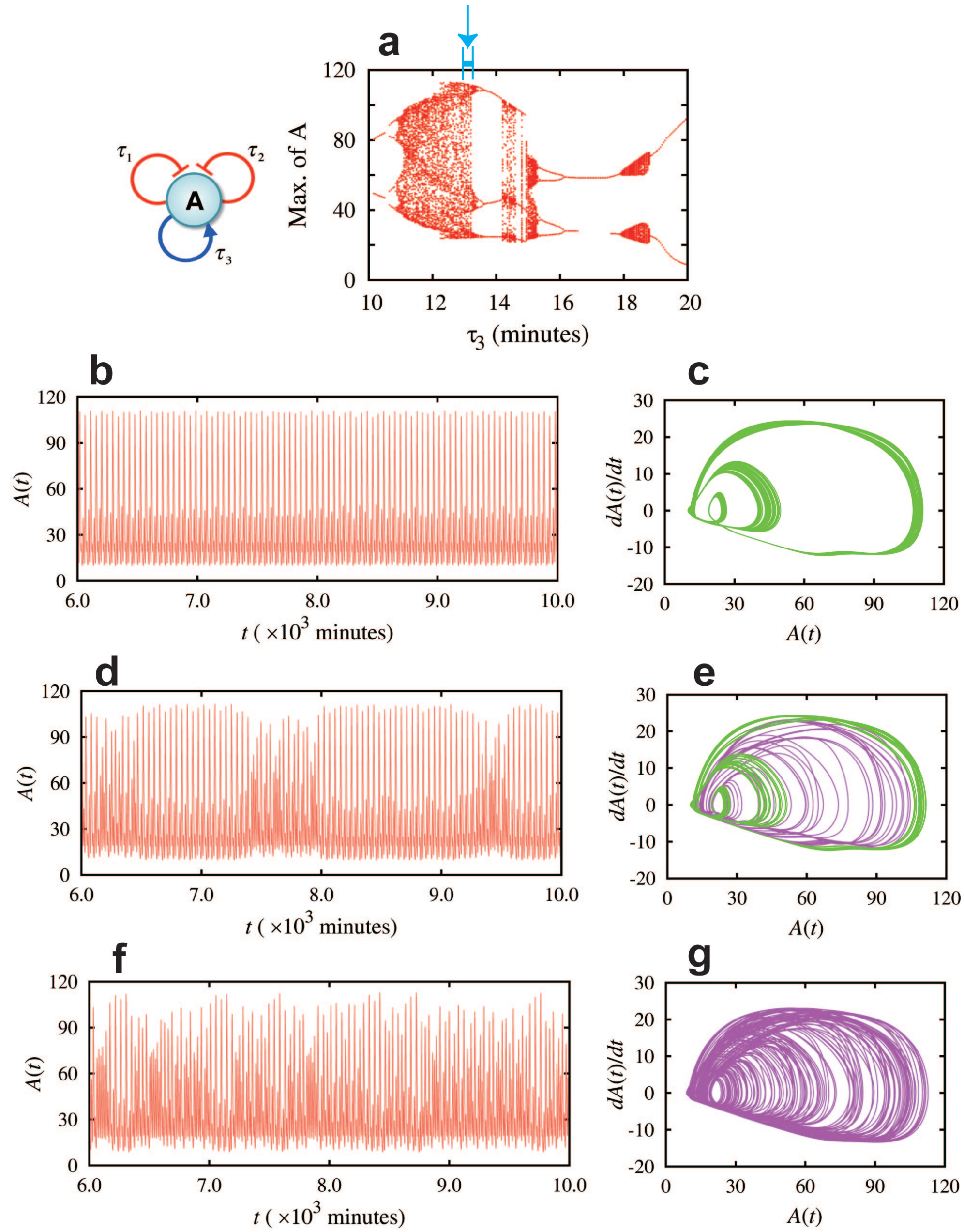

Figure SI5.1: **Intermittent dynamics between weak and strong chaotic modes for the one-gene element with two self-inhibitions and one self-activation.** Panel (a) is the bifurcation diagram of maximum levels of protein A with respect to τ_3, while fixing time delays $\tau_1 = 18$ minutes and $\tau_3 = 8.0$ minutes. The transition occurs from SC (panels (b) and (c)) to WC (panels (f) and (g)) dynamics around $\tau_3 = 13$ minutes (A blue line with bar pointed by a blue arrow in (a)). The intermittency between the two modes is observed where the values of τ_3 are between (panels (d) and (e)). Panels (b), (d), and (f) show the time trajectories for cases when the time delays of the self-activation $\tau_3 = 13.3$ minutes, 13.2 minutes, and 12.5 minutes respectively. Panels (c), (e), and (g) show the corresponding phase-space maps $A(t)$ - $dA(t)/dt$.

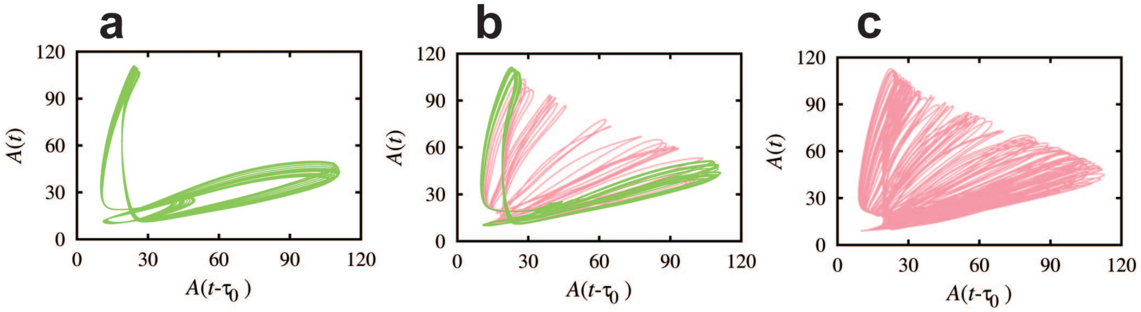

Figure SI5.2: **Phase-space maps $A(t-\tau_0)$ vs. $A(t)$ for the intermittent dynamics corresponding to the cases shown in Fig. SI5.1.** The phase space maps (a), (b), and (c) correspond to the time trajectory of (b), (d), and (f) in Fig. SI5.1 respectively. The values of τ_0 are set to be 14.80 minutes for (a), 15.37 minutes for (b), and 15.18 minutes for (c). The corresponding frequency for $\tau_0 = 15$ minutes is 0.067 /minutes. This frequency is found as a strong peak in the full spectra in Fig. SI5.3(a), (c), and (e). The panels (a), (b), and (c) indicate non-periodic dynamics. The panel (b) shows coexistence of the behaviors in both (a) (green) and (c) (red).

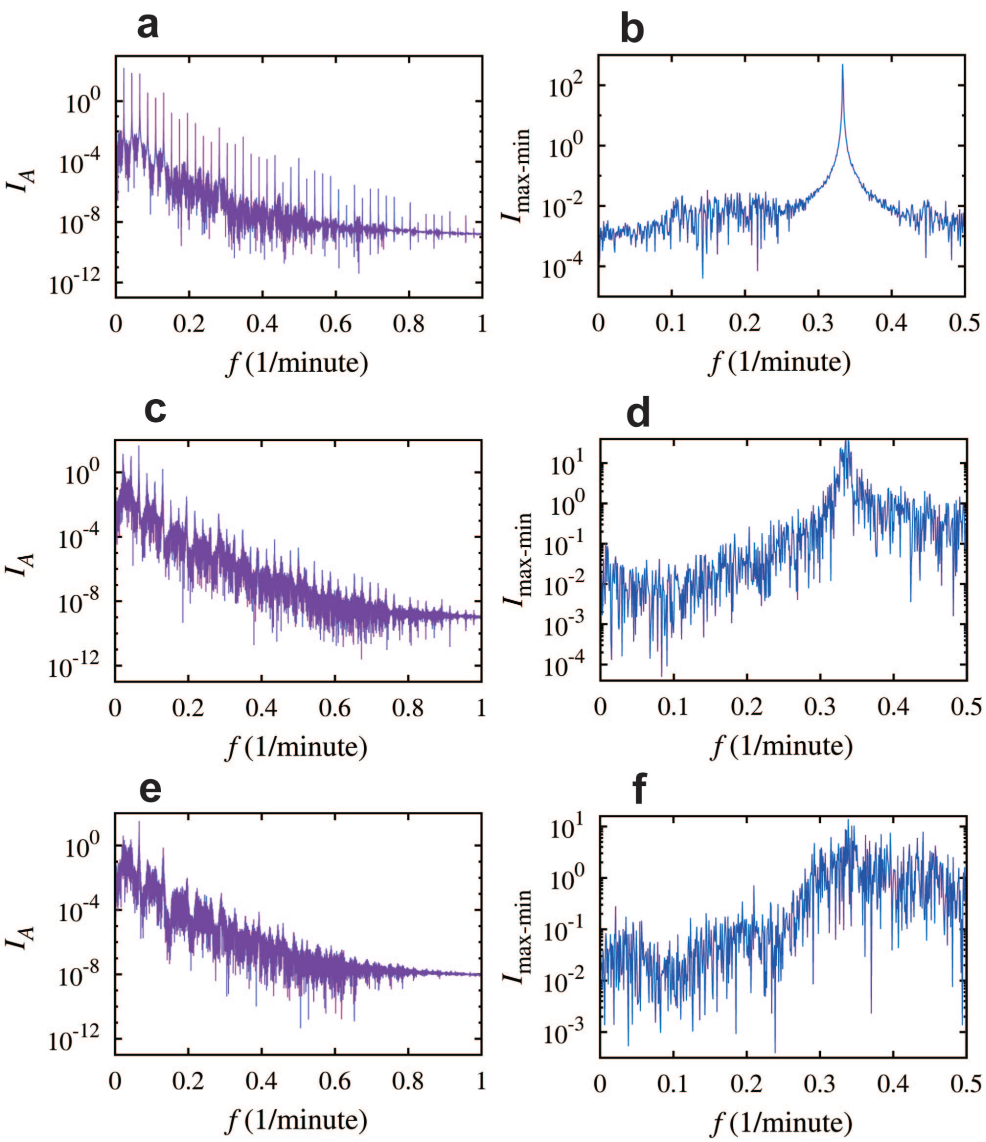

Figure SI5.3: **Full spectra and maximum-minimum spectra for the intermittent dynamics corresponding to the cases in Fig. SI5.1.** Left panels show the full spectra and right panels show the maximum-minimum spectra. The first row shows the spectra of the weak chaotic dynamics in the panels (b) and (c) in Fig. SI5.1. The third row shows to the spectra of the strong chaotic dynamics in the panels (f) and (g) in Fig. SI5.1. The second row shows the spectra of the WC-SC intermittent dynamics in the panels (d) and (e) in Fig. SI5.1.
